# Fungal and bacterial communities of ‘Pinot noir’ must: effects of vintage, growing region, climate, and basic must chemistry

**DOI:** 10.7717/peerj.10836

**Published:** 2021-02-04

**Authors:** Kerri L. Steenwerth, Ian Morelan, Ruby Stahel, Rosa Figueroa-Balderas, Dario Cantu, Jungmin Lee, Ron C. Runnebaum, Amisha T. Poret-Peterson

**Affiliations:** 1 Crops Pathology and Genetics Research Unit, USDA Agricultural Research Service, Davis, CA, United States of America; 2 Department of Viticulture and Enology, University of California, Davis, Davis, CA, United States of America; 3 Horticultural Crops Research Unit, USDA Agricultural Research Service, Parma, ID, United States of America; 4 Department of Chemical Engineering, University of California, Davis, Davis, CA, United States of America

**Keywords:** *Vitis vinifera* L., Environmental filtering, Distance-decay relationship, Microbiome, Wine grape, Enterobacteriaceae, *Hanseniaspora uvarum*, Grape must, Biogeography, Vintage

## Abstract

**Background:**

The geographic and temporal distributions of bacterial and fungal populations are poorly understood within the same wine grape cultivar. In this work, we describe the microbial composition from ‘Pinot noir’ must with respect to vintage, growing region, climate, and must chemistry across the states of California and Oregon, USA.

**Materials and Methods:**

We sampled ‘Pinot noir’ clone 667 clusters from 15 vineyards existing in a latitudinal gradient spanning nearly 1,200 km in California and Oregon for two vintages (2016 and 2017). Regions included five American Viticultural Areas (AVA). In order from southern California to Oregon, these AVAs were Santa Barbara, Monterey, Sonoma, Mendocino, and Willamette Valley. Uninoculated grape musts were subjected to 16S rRNA gene and ITS-1 amplicon sequencing to assess composition of microbial communities. We also measured grape maturity metrics. Finally, to describe regions by precipitation and growing degree days, we queried the Parameter-elevation Regressions on Independent Slopes Model (PRISM) spatial climate dataset.

**Results:**

Most of the dominant bacterial taxa in must samples were in the family *Enterobacteriaceae,* notably the lactic acid bacteria or the acetic acid bacteria groups, but some, like the betaproteobacterial genus *Massilia,* belonged to groups not commonly found in grape musts. Fungal communities were dominated by *Hanseniaspora uvarum* (*Saccharomycetaceae*). We detected relationships between covariates (e.g., vintage, precipitation during the growing season, pH, titratable acidity, and total soluble solids) and bacterial genera *Gluconobacter* and *Tatumella* in the family *Enterobacteraceae, Sphingomonas* (*Sphingomonodaceae*)*, Lactobacillus* (*Lactobacillaceae*), and* Massilia* (*Oxalobacteraceae*), as well as fungal genera in *Hanseniaspora, Kazachstania*,* Lachancea*, *Torulaspora* in the family *Saccharomycetaceae*, as well as *Alternaria* (*Pleosporaceae*)*, Erysiphe* (*Erysiphaceae*)*,* and* Udeniomyces* (*Cystofilobasidiaceae*)*.* Fungal community distances were significantly correlated with geographic distances, but this was not observed for bacterial communities. Climate varied across regions and vintages, with growing season precipitation ranging from 11 mm to 285 mm and growing degree days ranging from 1,245 to 1,846.

**Discussion:**

We determined that (1) bacterial beta diversity is structured by growing season precipitation, (2) fungal beta diversity reflects growing season precipitation and growing degree days, and (3) microbial differential abundances of specific genera vary with vintage, growing season precipitation, and fruit maturity metrics. Further, the correlation between fungal community dissimilarities and geographic distance suggests dispersal limitation and the vineyard as a source for abundant fungal taxa. Contrasting this observation, the lack of correlation between bacterial community dissimilarity and geographic distance suggests that environmental filtering is shaping these communities.

## Introduction

Regional and vintage variation in wine characteristics are important considerations in the production and marketing of wines. Spatiotemporal climatic variability, soil characteristics, and vineyard management practices are well understood to directly influence the physiology of grapevines and impact grape chemistry (Van Leeuwen & Destrac-Irvine, 2017). However, the effects of these factors on grapevine physiology do not fully explain regional and vintage variation in wine characteristics, and a growing body of evidence suggests that there may be a microbial component to this regional variability (Barata, Malfeito-Ferreira & Loureiro, 2012; Belda et al., 2017). Grape berries harbor diverse microbial communities, and some of these microbes have been detected in grape musts (Barata, Malfeito-Ferreira & Loureiro, 2012). However, the environmental sources and factors influencing grape must microbial communities are still poorly understood.

Numerous studies have described microbial communities in environments presumed to be sources of pre-inoculation grape must communities (Belda et al., 2021), but a coherent framework to describe processes shaping microbial diversity in grape musts remains elusive. Soil-borne microbial communities in vineyards are sensitive to management and exhibit spatial variation within the region of Napa Valley, CA, USA (Burns et al., 2015). Soils serve as a reservoir for microbial diversity and have been hypothesized to be a source of grape must microbial communities, but their overall similarity to grape must communities was notably low in ‘Merlot’ (Zarraonaindia et al., 2015). As with soils, ‘Sangiovese’ and ‘Dolcetto’ grapevine barks hosts a great diversity of microbes that vary regionally and respond to vineyard management; however, the similarity of these communities to grape must communities is also low (Vitulo et al., 2019). Some studies have observed vintage and cultivar effects in ‘Cabernet Sauvignon’, ‘Chardonnay’, and ‘Zinfandel’ grape musts, and ‘Merlot’ grapes (and other parts of the grapevine), suggesting a temporal and cultivar component to microbial diversity in vineyards (Bokulich et al., 2013; Bokulich et al., 2014; Zarraonaindia et al., 2015).

It is challenging to predict the composition of microbial grape must communities given the complexity of the problem, but some trends are present: the bacterial family *Enterobacteriaceae,* acetic acid bacteria (*Acetobacteraceae)*, and lactic acid bacteria are prevalent and abundant in many studies (Belda et al., 2017). The non-*Saccharomyces* yeast *H. uvarum* is commonly detected in grape musts worldwide (Barata, Malfeito-Ferreira & Loureiro, 2012). Some additional yeasts found naturally in grape musts including the genera *Metschnikowia, Torulaspora, Hanseniaspora, Pichia,* and *Lachancea* are being developed to meet increased consumer demand for distinctive wines (Masneuf-Pomarede et al., 2016). However, these well characterized taxa represent a minor fraction of the diversity encompassed by grape must microbial communities, and the environmental and regional factors influencing their abundances in wine grape musts are only beginning to be revealed.

A substantial effort has begun to describe regional variability of grape must microbial communities of many cultivars (Albertin et al., 2016; Barata, Malfeito-Ferreira & Loureiro, 2012; Belda et al., 2017; Bokulich et al., 2014). Additional work will contribute to better understanding of ‘Pinot noir’ must microbiomes across the western United States, a region where this cultivar has remained economically important. In California and Oregon combined, recent ‘Pinot noir’ grape production was worth ∼$425 million (California Department of Agriculture, 2020; OregonWineBoard, 2020). The objectives of this study were to describe the microbiome of pre-inoculation ‘Pinot noir’ grape must and examine possible links among region, climate variables, fruit maturity metrics, and microbial taxa. We aimed to account for these factors that are known to influence grape must microbiota in order to isolate the effects of climate and vintage. As such, we sampled from vines of the same scion clone managed by one commercial enterprise, and we standardized the processing protocol within a single research winery. We hypothesized that grape must microbial community structure would be linked to vintage and region, and that must bacterial communities would be dominated by *Acetobacteraceae* and lactic acid bacteria such as *Lactobacillaceae*.

## Materials and Methods

### Grape collection and processing

**Figure 1:**
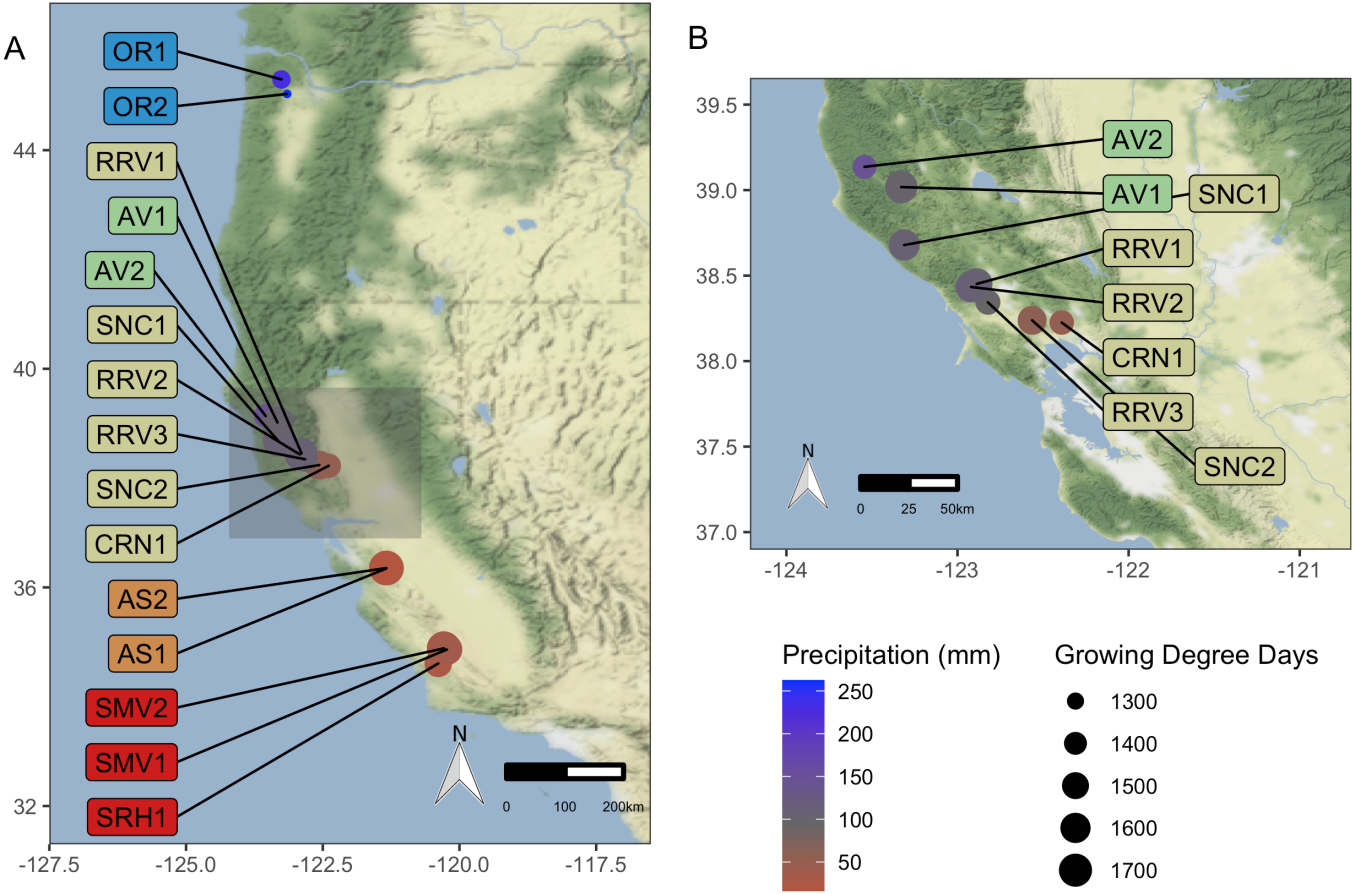
Vineyard Locations. ‘Pinot noir’ grapes were harvested from thirteen vineyards representing four AVAs in California and two vineyards in Oregon representing one AVA (total of 15). These vineyards were distributed across a 10.69 degree (1,187 km) latitudinal gradient. Grapes were transported to University of California, Davis (Davis, CA, USA) for processing and analysis. Size of vineyard points increases with mean GDD for 2016–2017, and color scale represents mean growing season precipitation for 2016–2017, ranging from red for lower values to blue for higher values. (A) displays all vineyard locations, while (B) provides a zoomed-in view of the northern California AVAs. Vineyard code details are in Table 1. Map credit: OpenStreetMap, 2019. Licensed under Stamen Design CC BY 3.0.

‘Pinot noir’ wine grapes (*Vitis vinifera* L.) were harvested in 2016 and 2017 from 15 commercial vineyards in California and Oregon, USA (Fig. 1). Vineyards were situated along a 10.69 degree (1,187 km) latitudinal gradient, representing five American Viticultural Areas (AVAs). AVAs are legal appellations designated for grape growing regions defined by the United States Tax and Trade Bureau, and US counties automatically designate legal appellation boundaries and names. The vineyards we sampled from were managed by a single commercial grower. We grouped vineyards by AVA (Santa Barbara County, CA, Monterey County, CA, Sonoma County, CA, Mendocino County, CA and Willamette Valley, OR) for analysis of regional trends. Samples were collected from one scion (clone 667) to eliminate cultivar effect on grape must microbiota (Bokulich et al., 2014; Zhang et al., 2019). Three vineyards (AV2, RRV2, and RRV3; see description of vineyard codes that is provided in Table 1) were planted with rootstock 3309C (*V. riparia* × *V. rupestris*), and two vineyards (OR1 and OR2) were planted with Riparia Gloire (*V. riparia* Michx). The other ten vineyards (AS1, AS2, AV1, CRN1, RRV1 SMV1, SMV2, SNC1, SNC2, and SRH1) were planted on rootstock 101-14 MGt (Millardet et de Grasset; *V. riparia* × *V. rupestris*).

**Table 1:** Vineyard information (year vines were planted, growing degree days, etc.) for the 15 sites and sample collection dates.

**Vineyard** **code**^a^	**AVA**^b^	**Year planted**	**GDD**^c^**2016**	**GDD** **2017**	**Precip.**^d^**(mm) 2016**	**Precip. (mm) 2017**	**Harvest date 2016**	**Harvest date 2017**
SMV1	Santa Barbara	2000	1,545	1,705	21	52	9∕8∕16	9∕4∕17
SMV2	Santa Barbara	2004	1,646	1,821	18	47	9∕8∕16	9∕4∕17
SRH1	Santa Barbara	n.d.^e^	1,437	1,589	19	35	9∕8∕16	9∕8∕17
AS1	Monterey	2005	1,454	1,684	12	21	8∕25∕16	8∕30∕17
AS2	Monterey	2005	1,680	1,846	12	28	8∕25∕16	8∕30∕17
CRN1	Sonoma	2012	1,400	1,472	28	77	9∕6∕16	8∕31∕17
RRV1	Sonoma	1998	1,576	1,707	52	165	9∕8∕16	9∕6∕17
RRV2	Sonoma	2000	1,556	1,681	63	167	9∕8∕16	9∕5∕17
RRV3	Sonoma	2006	1,400	1,497	55	132	9∕14∕16	9∕13∕17
SNC1	Sonoma	2000	1,582	1,670	60	157	9∕10∕16	9∕6∕17
SNC2	Sonoma	n.d.	1,498	1,617	37	81	8∕31∕16	8∕31∕17
AV1	Mendocino	2007	1,631	1,711	60	145	9∕6∕16	9∕12∕17
AV2	Mendocino	2000	n.c.	1,422	n.c.^f^	143	n.c.	9∕25∕17
OR1	Willamette Valley	2005	1,335	1,297	176	285	9∕16∕16	10∕4∕17
OR2	Willamette Valley	2004	n.c.	1,245	n.c.	262	n.c.	10∕4∕17

**Notes.**

a Vineyard code generated by the following abbreviations and followed by a number: SMV (Santa Maria Valley), SRH (Santa Rita Hills), AS (Arroyo Seco), CRN (Carneros), RRV (Russian River Valley), SNC (Sonoma Coast), AV (Anderson Valley), and OR (Willamette Valley, OR).

b American Viticultural Area (AVA).

c Growing Degree Days (GDD); calculated from 1 April–31 October in Celsius units with a baseline of 10 °C.

d Precipitation (Precip.); calculated from 1 April–30 September.

e n.d.: No data.

f n.c.: Not collected due to corresponding missing grape must samples.

Vineyard characteristics including AVA, year of planting, growing degree days (GDD), growing season precipitation, and harvest dates can be found in Table 1. Additional information about vineyard characteristics including elevation, sub-appellation, soil taxonomy (order and subgroup), soil texture, and grape must characteristics including total soluble solids (^∘^Brix), pH, and titratable acidity (g tartaric acid L^−1^) can be found in Table S1. Must samples were not collected from OR2 or AV2 in 2016 due to circumstances outside the purview of the authors. Grapes were harvested on different dates (Table 1), with the commercial producer aiming for total soluble solids of ∼24 °Brix. After arriving at the research winery (University of California, Davis, CA, USA), grapes in each batch were destemmed and split into four technical replicates. Equipment was sanitized and rinsed between batches. Must replicates were placed in sanitized 200 L stainless steel fermenters and maintained at 7 °C for three days prior to sterile collection of must for microbial analysis to identify vineyard microbial communities most relevant to fermentation (Bokulich et al., 2014). Grape must samples were stored in 15 mL conical vials in a −80 °C freezer until DNA extraction and amplicon sequencing. To evaluate fruit maturity, we measured grape must pH using a Thermo Scientific ORION 5 STAR (Waltham, MA, USA), titratable acidity (TA; g tartaric acid L^−1^) using a Mettler-Toledo DL50 autotitrator (Columbus, OH, USA), and total soluble solids (TSS; ^∘^Brix) using an Anton Paar DMA35 density meter (Graz, Austria).

### Climate data

We queried the Parameter-elevation Regressions on Independent Slopes Model (PRISM) 4 km dataset (Daly et al., 2008) to approximate daily high and low temperatures for the growing season, defined to be 1 April to 31 October (Winkler et al., 1974). To assess macroclimatic differences between regions, we estimated the sum of GDD for each vineyard from April 1st to October 31st, using the average of daily high and low temperatures and a baseline of 10 °C: Σ ((T_max_+T_min_)/2–10 °C) (Winkler et al., 1974). We also queried the PRISM dataset to obtain growing season precipitation estimates from April 1st to September 30th. We included October in GDD calculations given that this range is used to calculate GDD in the western US (Jones et al., 2010), but we excluded it in growing season precipitation measurements because the latest harvest date in our dataset was October 4th and we aimed to estimate the exposure of fruit to precipitation for this metric (Table 1).

### DNA Isolation and PCR

DNA was isolated from two mL of grape must using a 24:1 chloroform:isoamyl alcohol extraction protocol modified from Pereira, Guedes-Pinto & Martins-Lopes (2011) and Savazzini & Martinelli (2006). A Qubit dsDNA high sensitivity kit (Thermo Fisher Scientific Inc., Waltham, MA, USA) was used to quantify DNA concentration after extraction. DNA was amplified using GoTaq polymerase (Promega M300A; Promega Corp., Madison, WI, USA). For bacterial community composition, we amplified the V4 region of the 16S rRNA gene using forward-barcoded 515F –806R primers (Caporaso et al., 2011). PCR was conducted in a thermal cycler for 30 cycles under the following settings: denature at 94 °C for 45 s, anneal at 55 °C for 60 s, and extend at 72 °C for 90 s. These cycles were preceded by a 3-minute 94 °C denaturing step and followed by a 10-minute final extension step, then held at 4 °C after completion. We also used forward-barcoded BITS-B58S3 primers that target ITS region 1 for fungal community composition (Bokulich & Mills, 2013). PCR was performed for 30 cycles under the following settings: denature at 95 °C for 30 s, anneal at 55  °C for 30 s, and extend at 72 °C for 60 s. These cycles were preceded by a 2-minute 95  °C denaturing step and followed by a 5-minute final extension step, then held at 4 °C after completion. PCR products were validated by gel electrophoresis and cleaned using AMPure XP beads (Beckman Coulter A63880; Beckman Coulter, Brea, CA, USA) before pooling into libraries at equimolar concentrations. We also conducted PCR and sequencing on DNA isolation kit blanks to detect potential contaminating microbes.

### 16S rRNA gene and ITS-1 Amplicon Sequencing and Pre-processing

Amplicons were sequenced on the Illumina MiSeq platform using 2 × 250 bp paired-end reads (UC Davis DNA Technologies Core, Davis, CA, USA). We demultiplexed and trimmed barcodes from the raw amplicon sequencing data using Sabre and cutadapt (Martin, 2011). The scripts “01_demux_with_sabre.sh” and “02_remove_barcodes_with_cudapt.sh” include the exact parameters we used and are publicly available in our code repository (see Data Availability statement). We used the R package DADA2 to identify Amplicon Sequencing Variants (ASVs) and remove chimeric sequences in the 16S rRNA gene and ITS data (Callahan et al., 2016). The script “03_dada.R” includes the parameters we used to resolve ASVs. We used the DADA2 implementation of the Wang naïve Bayesian classifier with a modified SILVA 132 reference database to classify ASVs to the genus level using an 80% bootstrap value as a cutoff for assignment (Callahan, 2018; Wang et al., 2007). Finally, we implemented the approach described by Davis et al. (2018) to identify and remove potentially contaminating ASVs from our dataset. We used the phyloseq function “merge_samples” to merge technical replicates; this function sums reads from each sample and averages metadata values.

### Core microbiome, diversity, and differential abundance analyses

To gain a high-level overview of the taxonomic composition of the grape musts, we first identified a core microbiome of ASVs with relative abundances above 0.01% in at least 90% of the samples. We also conducted core microbiome analyses on each vintage separately. While useful to describe the general composition of grape must communities, this approach overlooks some abundant but not highly prevalent taxa. To identify these taxa, we filtered the ASV table to include non-core taxa whose relative abundances were above 1% in at least 5% of samples. Relative abundance measurements are heavily biased by factors like DNA extraction and differential amplification efficiency between taxa (McLaren, Willis & Callahan, 2019). To acknowledge these biases, we report relative abundance estimates as uncorrected relative abundances (URA).

To assess sequencing efforts, we conducted rarefaction analysis on alpha diversity metrics (richness, exponential Shannon, and inverse Simpson), extrapolating these values using the approach described by Chao et al. (2014) (Figs. S1 and S2). To evaluate alpha diversity, we first rarefied samples to even depth (16S dataset: 12,563 reads, ITS-1 dataset: 59,863 reads). We then estimated Hill diversities of order 0 (equivalent and hereafter referred to as “richness”), order 1 (equivalent and hereafter referred to as “exponential Shannon index”) and order 2 (equivalent to and hereafter referred to as “inverse Simpson index. We used both Bray–Curtis and Hellinger distances to quantify beta (between-sample) diversity. Specifically, we conducted Mantel tests (using Spearman’s rank correlation rho as the test statistic) to identify correlations between Hellinger distances and geographic distances. We conducted these analyses separately for each vintage because two vineyards were not sampled in 2016. We conducted permutational analyses of variance (PERMANOVAs), which implements a pseudo-F ratio suitable for unbalanced experimental design (McArdle & Anderson, 2001), to test for nonequivalence of centroids and dispersion of Bray Curtis dissimilarities grouped by AVA and vintage. We used non-metric multidimensional scaling (NMDS) to visualize differences between samples, mapping AVA and vintage onto the plots. Finally, we fitted environmental vectors to the ordination and used a permutation test (as implemented in the R function vegan::envfit) to assess the associations between beta diversity coordinates and fruit maturity metrics and climate variables. The relationship between microbial community structure and growing season precipitation was visualized by plotting precipitation as a contours over the NMDS ordination using the R function vegan::ordisurf.

We used the R package “Count Regression for Correlated Observations with the Beta-Binomial” (corncob) (Martin, Witten & Willis, 2020) to model abundances of microbial genera and test for statistical associations between microbial genera and variables of interest. Unlike other popular packages used to test for differential abundances, such as DESeq2 and edgeR, corncob was specifically designed to model microbial abundances and test hypotheses about the effects of covariates on relative abundances (Love et al., 2014; Robinson et al., 2010). This approach is especially tolerant to high variability in relative abundances, variability in sample depth, and the absence of taxa in samples—all common features of amplicon sequencing datasets. We modeled the abundances of bacterial and fungal genera with mean uncorrected relative abundances over 0.1%. We first tested for the effect of vintage, comparing a reduced model including AVA to a full model adding vintage using a likelihood ratio test. Other tests included AVA and vintage in the reduced model, which we compared to full models adding a fruit maturity metric or growing season precipitation. The beta-binomial regression coefficients (BBCE) that we report estimate the increase or decrease in logit-transformed relative abundance of a genus between two samples with one unit change in the covariate (Hailpern & Visintainer, 2003). Therefore, these coefficients cannot easily be compared across covariates given that some covariates spanned different ranges. For example, pH values only ranged from 3.4 and 3.8, while growing season precipitation ranged from 11 mm to 285 mm; it follows that if both covariates affected the abundance of a genus to the same extent over their respective ranges, we would expect greater values for beta-binomial regression coefficients for each unit change of pH compared to precipitation. We confirmed the better fit of significant beta binomial abundance full models by running simulations of reduced and full abundance models of individual genera 1,000 times. We then plotted 95% prediction intervals of the model outputs and visually confirmed that the full models were better fit to the data as indicated by narrower prediction intervals while still being consistent with the observed abundances. The code we used to visualize and assess models can be found in project repository listed in the Data Availability section. We controlled for Type I errors using the Benjamini–Hochberg procedure with a significant cutoff of 0.05 (Benjamini & Hochberg, 1995). However, due to our low sample size and slightly unbalanced design, these tests may have higher Type I error rates than FDR corrected for, so we acknowledge caution in the interpretation of *q*-values generated by this analysis. We plotted URAs of taxa associated with vintage, growing season precipitation, and fruit maturity metrics.

**Figure 2:**
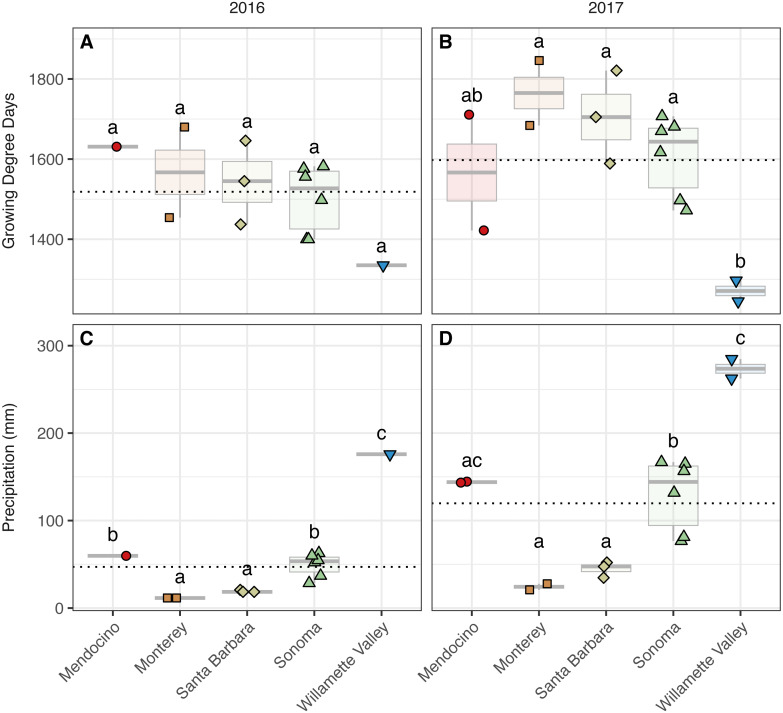
Growing degree days and precipitation grouped by AVA and vintage. Growing degree days grouped by AVA for the 2016 vintage (A) and 2017 vintage (B); growing season precipitation, grouped by AVA, for the 2016 vintage (C) and 2017 vintage (D). Dotted horizontal lines represent the corresponding vintage mean. Boxes followed by a different lowercase letter indicate significant differences (*p* < 0.05).

### Identifying differences between vintages and among AVAs

Effect of vintage and AVA within vintage was examined for climate variables, fruit maturity metrics, and alpha diversity. We implemented a linear mixed model (lme4; degrees of freedom method by Kenward-Roger) with main effects of ‘AVA’ and ‘vintage’, an interaction of ‘AVA × vintage’, and ‘vineyard’ as a random effect (*α* = 0.05) (Bates et al., 2015). Models were compared using chi-squared (*χ*^2^) statistics to portray differences in deviance between models and determine *p-* values (*α* = 0.05) based on likelihood ratio test comparisons (Bates et al., 2015). Multiple comparisons were determined using contrasts and Tukey’s test for mean separation (Table S2 for complete analyses), and significant differences are presented in the results. All variables were tested for normality (Shapiro–Wilk test) and homogeneity of variances (Levene’s test). Bacterial diversity measures (exponential Shannon values; inverse Simpson values) and fungal richness values were log-transformed to meet the normality criterion. Figures and text show untransformed values.

## Results

### Trends in climate and fruit maturity metrics

We detected trends among AVAs within vintage in both GDDs (AVA × vintage; *χ*^2^ = 31.32, *p* <  0.001) (Fig. 2, Table S2) (Fig. 2). In 2016, GDD exhibited a trend with respect to AVA: Santa Barbara (1,437–1,646), Monterey (1,454–1,680), Sonoma (1,400–1,582), and Mendocino (1,631) tended to have greater GDD than the northernmost Willamette Valley vineyard (1,335) (not significant, ‘NS’). Similarly, GDD in 2017 differed significantly by AVA (*p* <  0.05) and decreased from the southernmost AVA to the northernmost AVA: Santa Barbara (1,589–1,821), Monterey (1,684–1,846), Sonoma (1,472–1,707), and had greater GDD than the Willamette Valley (1,245–1,297), but Mendocino (1,422–1,711) was not significantly different from any AVA. Mean comparisons between vintages within AVA were significant for all comparisons (*p* <  0.01) except Willamette Valley, revealing the increase in GDD by AVA from 2016 to 2017.

Growing season precipitation also differed between by AVA within vintage (AVA × vintage: *χ*^2^ = 11.89, *p* <  0.05) (Fig. 2, Table S2). Precipitation in 2016 increased from the southernmost AVA to the northernmost AVA. Mean separation tests revealed that vineyards in Santa Barbara (18–21 mm) and Monterey (12 mm) received less precipitation than those in Sonoma (28–63 mm), Mendocino (60 mm), and the Willamette Valley (176 mm) (*p* <  0.01). Precipitation also differed among AVAs in 2017, and the same trend was identified (*p* <  0.01), except that mean growing precipitation in Mendocino and Willamette Valley did not differ. Mean comparisons between vintages within AVA were significant for all comparisons (*p* <  0.01), revealing the increase in growing season precipitation from 2016 to 2017 for all AVAs.

**Figure 3:**
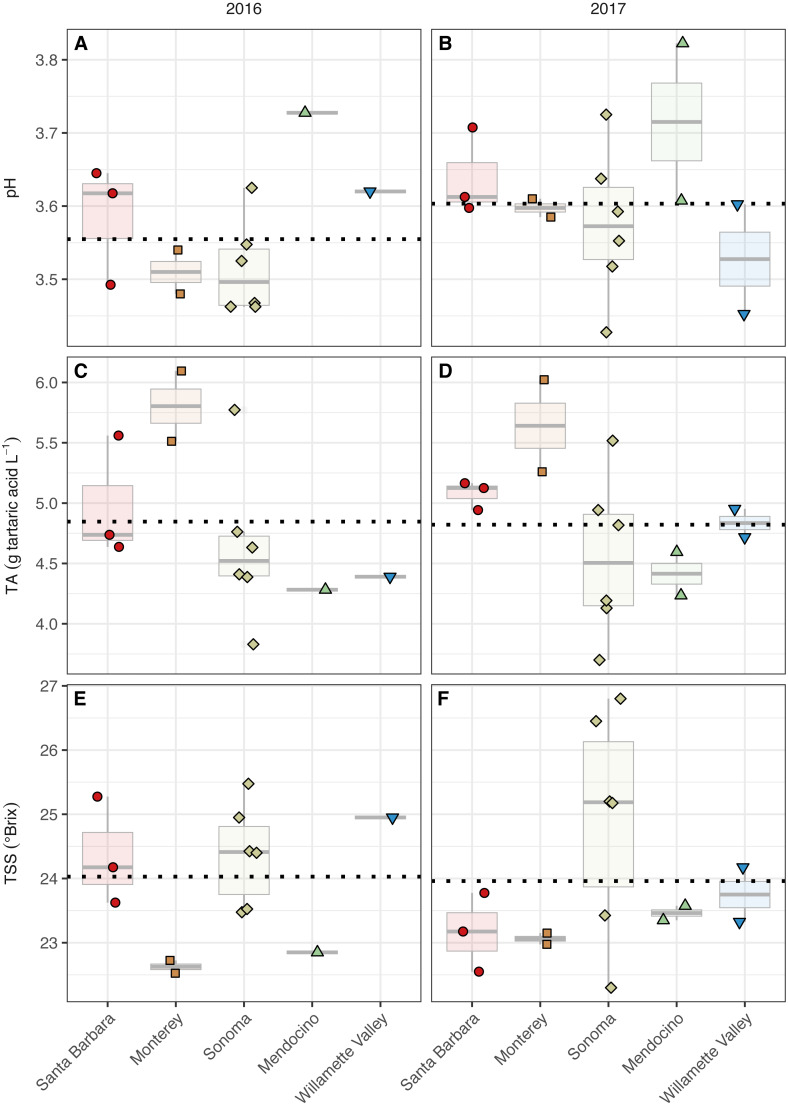
Grape maturity metrics grouped by AVA and vintage. Grape must pH (A–B), titratable acidity (g tartaric acid L^−1^) (C–D), and total soluble solids (°Brix) (E–F) of grape musts for the 2016 vintage (A, C, E) and the 2017 vintage (B, D, F). Dotted horizontal lines represent the corresponding vintage mean.

The grape must pH differed with respect to AVAs (AVA: *χ*^2^ = 9.98, *p* <  0.05) but not vintage. However, mean separation tests revealed only marginal insignificance between Mendocino and Sonoma (*p* = 0.08) (Fig. 3, Table S2). Sonoma, Mendocino, and Willamette Valley tended to have lower pHs than Santa Barbara and Monterey in both years. Grape must titratable acidity (TA) concentrations differed by AVA (AVA: *χ*^2^ = 11.83, *p* <  0.05) but not vintage. Primarily, Monterey had greater TA values than Sonoma (*p* <0.05), but no other differences among AVAs were detected. Total soluble solids (TSS) only differed by AVA (*χ*^2^ = 9.75, *p* <  0.05) but mean separation tests revealed only marginal insignificance between Monterey and Sonoma (*p* = 0.09). In 2016, Monterey (22.5–22.7°Brix) and Mendocino (22.9°Brix) tended to have lower TSS values than Santa Barbara (23.6–25.3°Brix), Sonoma (23.5–25.5° Brix), and Willamette Valley (25.0°Brix). In 2017, no trend in TSS was observed, though Sonoma (22.3–26.8°Brix) displayed a wider range in TSS than Santa Barbara (22.6–23.8°Brix), Monterey (23.0–23.2°Brix), Mendocino (23.4–23.6°Brix), and Willamette Valley (23.3–24.2°Brix). No significant effect of AVA × vintage was detected for any basic maturity metric.

### The core must microbiome is made up of *Proteobacteria* and *H. uvarum,* while the variable microbiome is more taxonomically diverse

Eighteen core bacterial ASVs were present in both vintages and all AVAs, as defined by an uncorrected relative abundance (URA) above 0.01% in at least 90% of samples (Table S2A). Of these ASVs, all but one (in family *Microbacteriaceae*) were classified in the phylum *Proteobacteria.* The most abundant of these taxa were *Tatumella* (*Enterobacteracieae*)*,* with an average URA of 37% (highly abundant in the 2016 vintage), and *Massilia* (*Oxalobacteraceae*), with an average URA of 9% (highly abundant the 2017 vintage). By vintage, we identified 40 core bacterial ASVs in 2016 and 19 ASVs in 2017 (Table S2B and S2C). We also identified 25 variable ASVs (Table S2D) among all samples from both vintages, which we defined as having at least 1% URA in between 5% and 90% of samples. Like the core microbiome, the variable microbiome was dominated by *Proteobacteria*, but ASVs from *Actinobacteria* (genus *Janibacter*, family *Intrasporangiaceae*), and *Firmicutes* (genus *Lactobacillus,* family *Lactobacillaceae*) were also present.

All four core fungal ASVs (Table S3A) were classified as *H. uvarum* (*Saccharomycetaceae*). This single genus made up over 87% of fungal URAs. An unassigned ASV in genus *Alternaria* (*Pleosporaceae*) was also detected in over 90% of 2016 samples, with an average URA of 0.04% (Table S3B). In 2017, core ASVs were all classified as *H. uvarum*. The three fungal ASVs in the variable microbiome were assigned to *H. uvarum, Erysiphe necator* (*Erysiphaceae*)*,* and *Lachancea quebecensis* (*Saccharomycetaceae*) (Table S3D). *E. necator* was particularly abundant in samples from the 2017 vintage from CRN1 in Sonoma, with URAs from 90–95% in those samples. The median URA of *L. quebecensis* was 0.05%, and it reached up to 4.7% in the 2017 vintage from vineyard AV2 in Mendocino.

### Microbial diversity of grape must with respect to vintage and AVA

**Figure 4:**
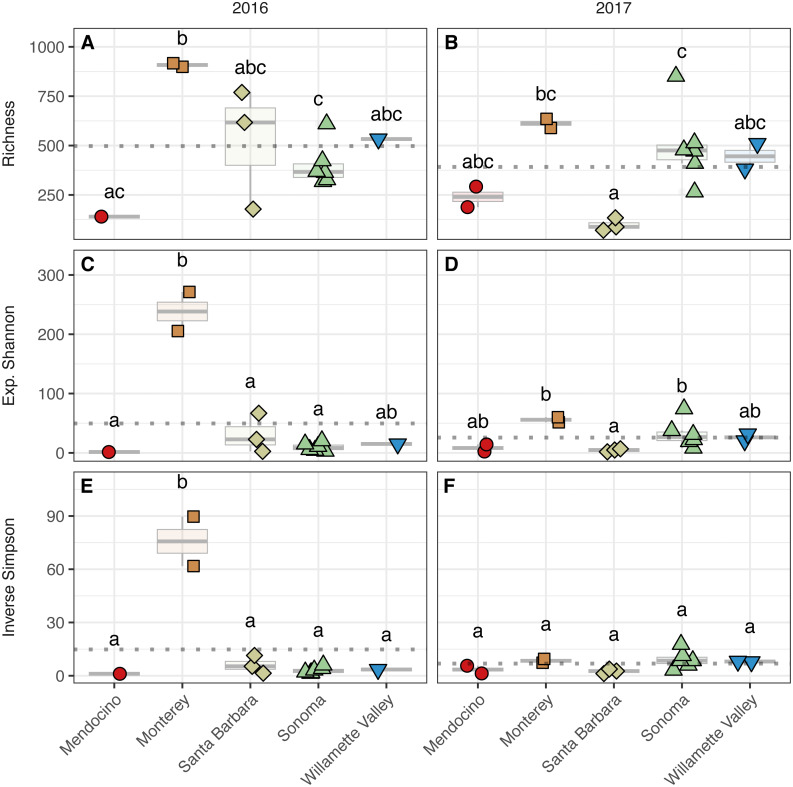
Bacterial alpha diversity. Bacterial community richness (A–B), exponential Shannon index (C–D), and inverse Simpson (E–F) index of grape musts for the 2016 vintage (A, C, E) and the 2017 vintage (B, D, F). Dotted horizontal lines represent the corresponding vintage mean. Boxes followed by a different lowercase letter indicate significant differences (*p* < 0.05).

**Figure 5:**
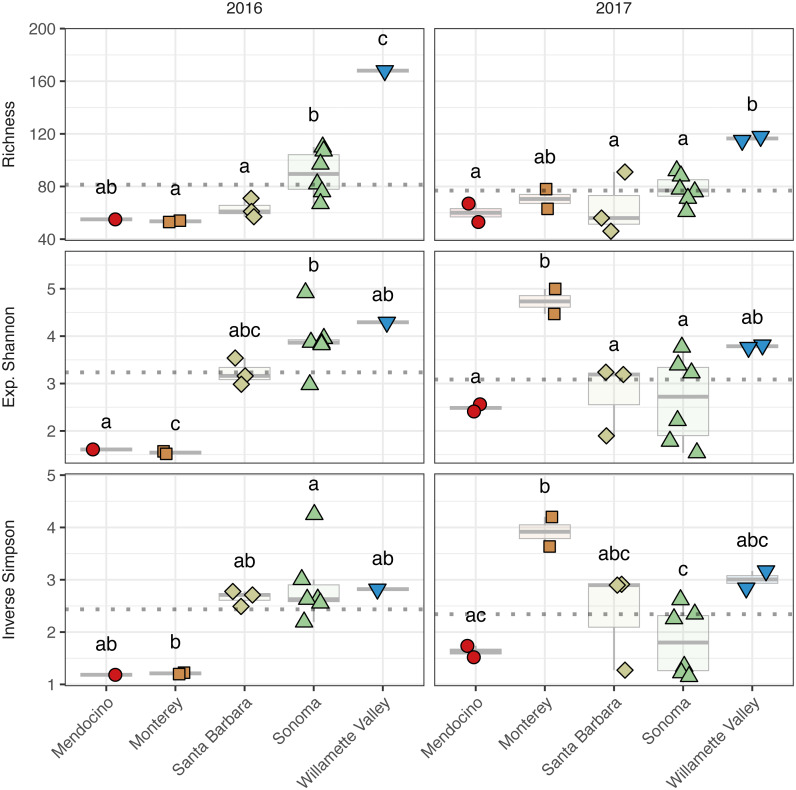
Fungal alpha diversity. Fungal community richness (A–B), exponential Shannon index (C–D), and inverse Simpson (E–F) index of grape musts for the 2016 vintage (A, C, E) and the 2017 vintage (B, D, F). Dotted horizontal lines represent the corresponding vintage mean. Boxes followed by a different lowercase letter indicate significant differences (*p* < 0.05).

To assess alpha diversity, we calculated richness, exponential Shannon, and Inverse Simpson indices (Figs. 4 and 5, Table S2). Exponential Shannon and Inverse Simpson indices were accurately estimated from our sequencing effort based on saturation of rarefaction curves for these estimates (Fig. S1A) however, curves for bacterial richness did not plateau, suggesting an underestimate in community richness. Bacterial richness differed by AVA within vintage (AVA × vintage, *χ*^2^ = 16.47, *p* <  0.01) (Fig. 4). This was also true for bacterial exponential Shannon values (AVA × vintage, *χ*^2^ = 16.04, *p*  <  0.01) and inverse Simpson values (AVA × vintage, *χ*^2^ = 25.17, *p*  < 0.0001). In 2016, only Monterey had greater bacterial richness than Sonoma (*p*  <  0.01) and Mendocino (*p* <  0.01). In 2017, bacterial richness was lower in Santa Barbara than Monterey (*p* <  0.01) and Sonoma (*p* <  0.01). For the bacterial exponential Shannon index in 2016, Monterey had greater values than Santa Barbara (*p* <  0.05), Sonoma (*p* <  0.001), and Mendocino (*p* <  0.001). In 2017, exponential Shannon index was lower in Santa Barbara than in Monterey (*p* <  0.05) and Sonoma (*p* <  0.05). For the bacterial inverse Simpson values in 2016, Monterey had greater values than all other AVAs (*p* <  0.01). In 2017, bacterial inverse Simpson values did not differ among AVAs. When examining changes in diversity metric by vintage within AVA from 2016 to 2017, Santa Barbara decreased in richness and the exponential Shannon index (*p* <  0.05), Sonoma increased in the exponential Shannon index (*p* <  0.05). Monterey decreased in the exponential Shannon index while Sonoma increased for inverse Simpson (*p* <  0.01).

Fungal alpha diversity was consistently lower than bacterial alpha diversity, was similar between vintages, and exhibited regional trends (Figs. 4 and 5, Table S2). Rarefaction analyses of fungal alpha diversity metrics yielded results similar to bacteria (Fig. S2). Fungal richness, exponential Shannon and inverse Simpson all differed by AVA within vintage (AVA × vintage: richness, *χ* ^2^ = 14.76, *p* <  0.01; exponential Shannon, *χ*^2^ = 31.20, *p* <  0.0001; inverse Simpson, *χ*^2^ = 26.52, *p* <  0.0001). Fungal richness in 2016 was greatest in Willamette Valley, followed by Sonoma, and then Santa Barbara and Monterey (*p* <  0.001). Fungal richness in Mendocino only differed from Willamette Valley (*p* <  0.001). In 2017, fungal richness also differed among AVAs. Willamette Valley once again had higher richness than Mendocino and Santa Barbara (*p* <  0.01), but differences among other AVAs was not detected. Fungal exponential Shannon values in Sonoma were higher than Monterey (*p* <  0.001) and Mendocino (*p* <0.05) in 2016, while Monterey had greater values than all other AVAs in 2017 (*p* <  0.05) (except Willamette Valley). Fungal inverse Simpson values were greater in Sonoma than Monterey in 2016 (*p* <  0.05), but in 2017, Monterey had greater values than Sonoma (*p* <  0.05) and Mendocino (*p* <  0.01). Willamette Valley had the highest richness in both vintages, and fungal alpha diversity among AVAs was characterized by relative consistency between the vintages except for Monterey (*p* <  0.05, richness and exponential Shannon), which tended to have an increase in richness and diversity between 2016 and 2017, and Sonoma, which decreased between vintages (*p* <  0.05, richness and inverse Simpson).

**Figure 6:**
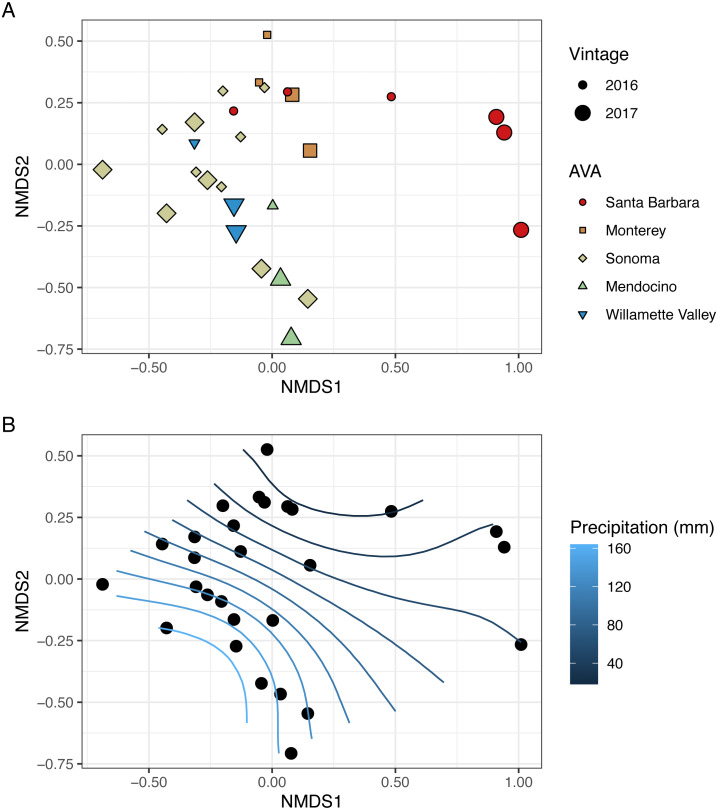
Bacterial beta diversity is linked to AVA, vintage, and precipitation. NMDS ordination plots (*k* = 3, stress = 0.10) visualize Bray Curtis distances. In (A), AVA corresponds with shape and color, and year corresponds with point size. In (B), the same ordination is displayed with precipitation mapped as a contour plot.

**Figure 7:**
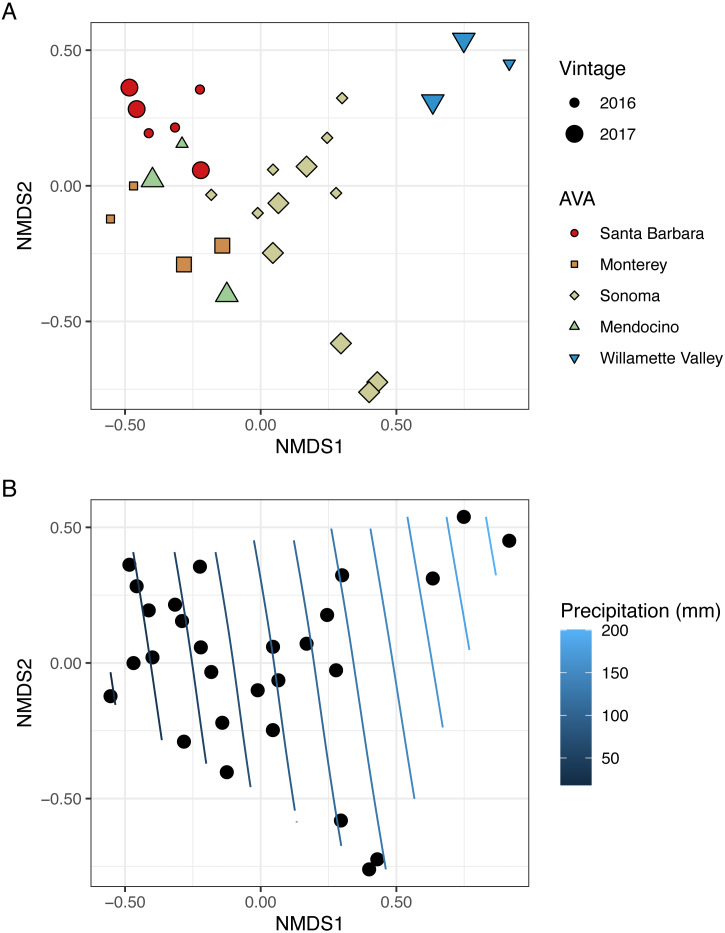
Fungal beta diversity is linked to AVA, vintage, and precipitation. NMDS ordination plots (*k* = 3, stress = 0.12) visualize Bray Curtis distances. In (A), AVA corresponds with shape and color, and year corresponds with point size. In (B), the same ordination is displayed with precipitation mapped as a contour plot.

To visualize beta diversity, we conducted nonmetric multidimensional scaling (NMDS) on bacterial (*k* = 3, stress=0.10) and fungal (*k* = 3, stress=0.12) Bray-Curtis dissimilarity matrices (Figs. 6A, 7A). Fungal and bacterial communities tended to cluster by AVA and, in some cases, by vintage. For example, bacterial communities from Santa Barbara tended to cluster to the right of the origin along axis NMDS1, especially in 2017 (Fig. 6A), whereas samples from AVAs with greater precipitation and fewer GDD ordinated to the left of the origin along axis NMDS1. Fungal communities from Willamette Valley and Sonoma AVAs clustered on the right of the origin along axis NMDS1 while the other AVAs clustered in the opposite direction along axis NMDS1.

We also fitted continuous metadata variables (total soluble solids, pH, titratable acidity, elevation, GDD, and growing season precipitation) onto these ordinations and used a permutation test to assess significance of the correlations between these variables and community structure (Table S4). Growing season precipitation significantly correlated with ordination coordinates of bacterial communities [*R*^2^ = 0.48; Envfit permutation test (EPT), *p* = 0.0009)] and fungal communities (*R*^2^ = 0.51; EPT, *p* = 0.0007). GDD was significantly correlated with fungal communities (*R*^2^ = 0.34, *p* = 0.005). Bacterial community structure correlated with pH (*R*^2^ = 0.37; EPT, *p* = 0.003), TA (*R*^2^ = 0.31; EPT, *p* = 0.009), and TSS (*R*^2^ = 0.23; EPT, *p* = 0.04) (Table S4A); however, fungal communities had no significant associations with grape must characteristics (Table S4A). Elevation did not correlate with either bacterial or fungal communities (Table S4A and Table S4B). We plotted a contour of the growing season precipitation gradient onto the NMDS plots to visualize how precipitation related to microbial bacterial and fungal beta diversity (Figs. 6B and 7B).

We conducted PERMANOVA using Bray–Curtis distance matrices to test for equality of centroids and dispersion when grouping samples by vintage or AVA. We detected significant differences in bacterial Bray–Curtis dissimilarities between vintages (*R*^2^ = 0.11; *p* = 0.004) and AVA (*R*^2^ = 0.31; *p* = 0.003). Fungal Bray-Curtis distances also differed between vintage (*R*^2^ = 0.12, *p* = 0.005) and AVA (*R*^2^ = 0.22, *p* = 0.045). We conducted a Mantel test to examine whether geographic distances correlated with Hellinger distances for each vintage. Bacterial Hellinger distances were not significantly correlated with geographic distances for either the 2016 (Spearman’s rho: 0.13, *p* = 0.25) or 2017 (Spearman’s rho: -0.15, *p* = 0.74) vintage. Fungal Hellinger distances, however, had significant correlations for both the 2016 (marginally) (Spearman’s rho: 0.39, *p* = 0.058) and 2017 (Spearman’s rho: 0.59, *p* = 0.004) vintages. In summary, grape must fungal communities were more similar if the vineyards were geographically close. While bacterial communities did exhibit regional patterns as revealed by a PERMANOVA, they did not exhibit this distance-decay relationship.

### Microbial taxa are associated with vintage, precipitation, and fruit maturity metrics

**Figure 8:**
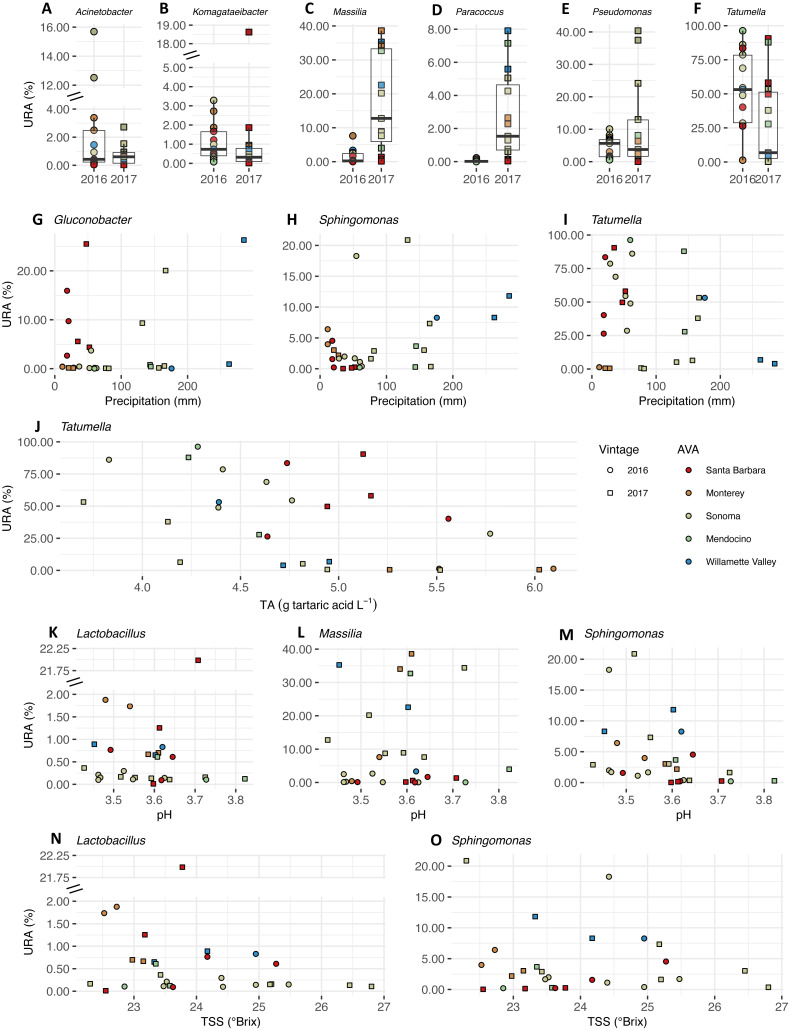
Bacterial differential abundance with respect to vintage, growing season precipitation, and fruit maturity metrics. Uncorrected relative abundances (percent URAs) of bacterial genera whose beta-binomial abundance models were significantly improved (as determined by a likelihood ratio test) by including (A–F) vintage as a covariate; (G–I) precipitation as a covariate; (J) titratable acidity (g tartaric acid L^−1^) as a covariate; (K–M) pH as a covariate; (N–O) total soluble solids (TSS; ° Brix) as a covariate. Note the breaks in the *y*-axis (A, B, K and N) to facilitate ease of viewing. Figure S3 shows this figure without *y*-axis breaks. Color of points indicates the respective AVA, as follows: Santa Barbara = red, Monterey = orange, Sonoma = yellow, Mendocino = green, Willamette Valley = blue. Symbols of vintage, as follows: 2016 vintage = circle, 2017 vintage = square.

We plotted the uncorrected relative abundances (URA) of bacterial genera whose beta binomial abundance models were significantly improved (R-package corncob, likelihood ratio test, FDR *q*-value<  0.05) by the addition of vintage, precipitation and fruit maturity metrics as parameters (Fig. 8). Beta binomial coefficient estimates (BBCE), standard errors, t-values, and *p*-values can be found in Table S6. These coefficients estimate the increase or decrease in logit-transformed relative abundance of a genus between two samples with one unit change in the covariate. Median URAs of *Acinetobacter* (2016: 0.4%, 2017: 0.6%)*, Massilia* (2016: 0.2%, 2017: 13%)*,* and *Paracoccus* (2016: 0.01%, 2017: 1.5%) increased from 2016 to 2017, while median abundances of *Komagataeibacter* (2016: 0.7%, 2017: 0.3%)*, Pseudomonas* (2016: 5.6%, 2017: 3.7%), and *Tatumella* (2016: 53%, 2017: 6.8%) decreased (Fig. 8A). URAs of *Gluconobacter* (BBCE = 0.03, *q* = 0.01) and *Sphingomonas* (beta-binomial regression coefficient = 0.01, *q* = 0.0003) were positively associated with growing season precipitation, while abundances of *Tatumella* (BBCE = −0.03, *q* = 0.003) had a slightly negative association with precipitation (Fig. 8B). Coefficient estimates suggest that *Tatumella* URAs decreased with increasing TA (BBCE = −1.4, *q* = 0.03) (Fig. 8C). *Massilia* (BBCE = −6.5, *q* = 0.0002) and *Lactobacillus* (BBCE = −2.9, *q* = 0.01) URAs exhibited negative associations with pH while *Sphingomonas* (BBCE = 1.6, *q* = 0.01) URAs exhibited a positive association with pH (Fig. 8D). *Lactobacillus* (BBCE = −0.07, *q* = 0.0004) and *Sphingomonas* (BBCE = −0.28, *q* = 0.0004) were negatively associated with TSS (Fig. 8E).

**Figure 9:**
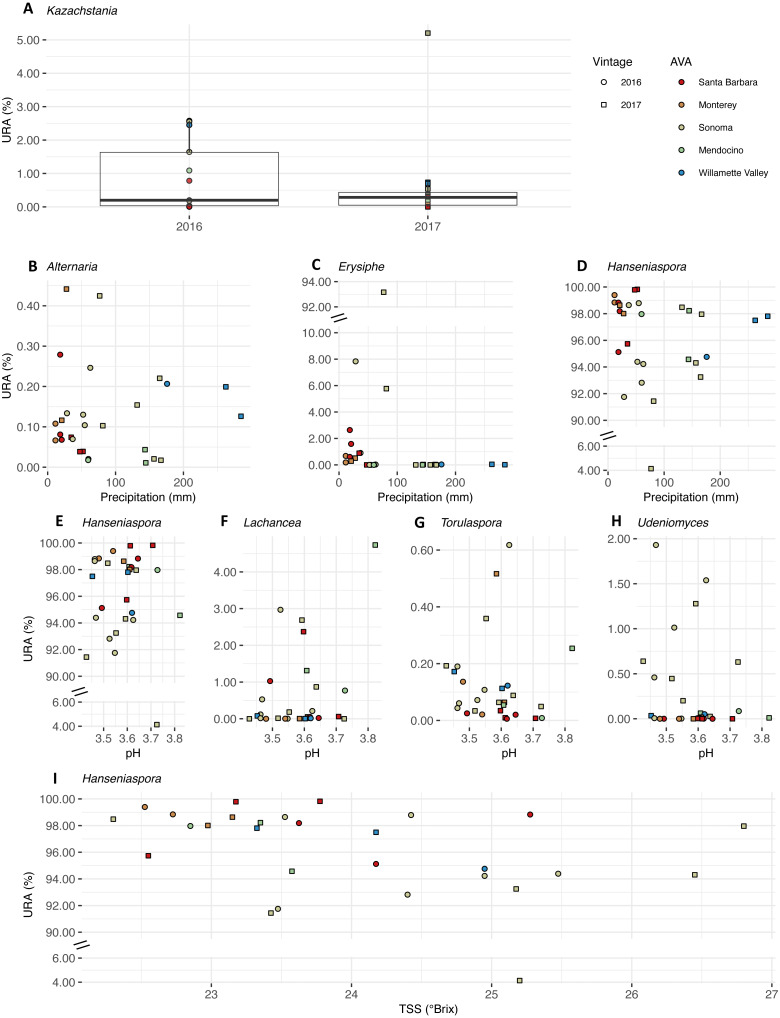
Fungal differential abundance with respect to vintage, growing season precipitation, and fruit maturity metrics. Uncorrected relative abundances (percent URAs) of fungal genera whose beta-binomial abundance models were significantly improved (as determined by a likelihood ratio test) by including (A) vintage as a covariate; (B–D) growing season precipitation as a covariate; (E–H) pH as a covariate; (I) total soluble solids (TSS) as a covariate. Note the breaks in the *y*-axis (C, D and E) to facilitate ease of viewing. Figure S4 shows this figure without *y*-axis breaks. Color of points indicates the respective AVA, as follows: Santa Barbara = red, Monterey = orange, Sonoma = yellow, Mendocino = green, Willamette Valley = blue. Symbols of vintage, as follows: 2016 vintage = circle, 2017 vintage = square.

We plotted the uncorrected relative abundances (URA) of fungal genera whose beta binomial abundance models were significantly improved (R-package corncob, likelihood ratio test, FDR *q*-value<  0.05) by the addition of vintage, precipitation, and pH and TSS as parameters (Fig. 9). TA was not plotted as it did not improve the model fit. Results of the beta-binomial regressions can be found in Table S5B. *Kazachstania* median URAs increased slightly from 2016 to 2017 (2016: 0.2%, 2017: 0.3%) (Fig. 9A). Abundances of *Hanseniaspora* (BBCE = 0.02, *q* = 0.004) exhibited a slightly positive association with precipitation, while *Erysiphe* (BBCE = −0.07, *q* = 1. 2 ×10^−8^)*,* and *Alternaria* (BBCE = −0.02, *q* = 0.008) exhibited slightly negative associations (Fig. 9B). Coefficient estimates indicate that *Lachancea* (BBCE = 6.1, *q* = 0.003) abundances increased with pH, while *Hanseniaspora* (BBCE = −5.2, *q* = 0.002)*, Udeniomyces* (BBCE = −5.0, *q* = 0.015)*,* and *Torulaspora* (BBCE = −2.5, *q* = 0.015) abundances decreased with increasing pH (Fig. 9C). Finally, *Hanseniaspora* abundances exhibited a slightly negative association with TSS (BBCE = −0.53, *q* = 0.008) (Fig. 9D).

## Discussion

### The ‘Pinot noir’ must microbiome is dominated by *Enterobacteriaceae* and *H. uvarum*

As studies specifically examining geographic distribution of ‘Pinot noir’ bacterial and fungal communities in grape must are limited, we include findings from other grape cultivars to provide context for our work. Grape must bacterial communities were dominated by *Enterobacteriaceae,* a large bacterial family partly defined by its capacity to ferment glucose, in both the 2016 and 2017 vintages*.* This family has previously been found in grape musts of ‘Grenache’ and ‘Carignan’ in Spain (Portillo et al., 2016) and in ‘Chardonnay’ from California, where its abundance altered with vintage (Bokulich et al., 2014). The 16S rRNA gene is a poor marker for genus-level classification of bacteria in this family, and the taxonomy of this family is still revised frequently (Alnajar & Gupta, 2017; Naum, Brown & Mason-Gamer, 2009). The most abundant and prevalent ASV in our dataset was assigned to the genus *Tatumella* and had an average URA of 37%. *Tatumella* bacteria have been found on both *Botrytis* free and infected ‘Mavroliatis’ and ‘Sefka’ grapes (Nisiotou et al., 2011). *Botrytis* infection was also linked to a higher population of *Tatumella* when compared to *Botrytis* free grapes in both cultivars (Nisiotou et al., 2011), but our samples do not contain *Botrytis cinerea* at abundances above 0.1%. *Tatumella* has not been previously described as an abundant member of the grape must microbiome, possibly due to the fact it is difficult to culture (Farmer, 2015) or the recent transfer of some *Pantoea* (*Enterobacteriaceae*) species to this genus (Brady et al., 2010). A non-*Enterobacteriaceae* bacterium of note was classified as the betaproteobacteria *Massilia* (*Oxalobacteraceae*), which had a striking abundance pattern with respect to vintage and pH. It was the most abundant bacterium in the 2017 vintage with a URA of 12%, but had a mean URA of only 0.1% in the 2016 vintage. In previous studies, *Massilia* has been detected on ‘Chardonnay’ and ‘Merlot’ grape berries (Leveau & Tech, 2011; Martins et al., 2013).

*Hanseniaspora uvarum* (*Saccharomycetaceae*) dominated the fungal communities that varied with region, vintage, and grape must characteristics. Here, *H. uvarum* showed a regional correspondence with precipitation, and TSS and pH in ‘Pinot noir’ grape must (Fig. 9). Intraspecific geographic structure of *H. uvarum* diversity (111 strains studied) has previously been observed (Albertin et al., 2016). Given the consistent regional trends in our single-winery study, our data suggest that the geographic diversity of this species extends beyond the winery back to the vineyard. Further, like Albertin et al. (2016), we detected a vintage effect; this suggests a notable and variable population ecology for this species. The genus *Hanseniospora* found in grape must has also been shown to directly vary with precipitation and humidity across a variety of grape cultivars (Jara et al., 2016). Interest in *H. uvarum* exists as some strains of *H. uvarum* (co-fermented with *S. cerevisiae*) increased positive attributes in wine (Tristezza et al., 2016). We also acknowledge that the temperature of the cold soak (ca. 4 °C) may have influenced the composition of the fungal community. For instance, Liu et al. (2020) recently reviewed work that revealed that a pre-fermentation cold-soak of red grape cultivars (‘Malbec’, ‘Cabernet Sauvignon’) at cooler temperatures (8 ±  1 °C) led to higher populations of *S*. *cerevisiae* whereas a warmer cold soak (14 ± 1 °C) encouraged growth of populations of *H*. *uvarum* and *Candida zemplinina* on the fifth day (Maturano et al., 2015). In our study, *H. uvarum* was a dominant member of the ‘Pinot noir’ fungal community after three days at a comparatively cooler pre-fermentation cold soak (ca. 4 °C).

### Associations between microbial genera and vintage, climate, and fruit maturity metrics

We detected a number of statistical associations between microbial genera and vintage, growing season precipitation, and fruit maturity metrics. Bacterial genera *Komagataeibacter* (*Acetobacteraceae*)*, Pseudomonas* (*Pseudomonadaceae*)*,* and *Tatumella* were associated with the 2016 vintage (Fig. 8, Table S6A). Genera *Acinetobacter* (*Moraxellaceae*)*, Massilia,* and *Paracoccus* (*Rhodobacteraceae*) were associated with the 2017 vintage. Bacterial genera *Gluconobacter* and *Sphingomonas* were positively associated with growing season precipitation, while *Tatumella* had a negative association. These vintage differences may correspond to a higher environmental moisture content and/or temperature given that the vineyards in this study had higher growing season precipitation and GDD in 2017 than in 2016. Revealing a likely sensitivity to environmental gradients, *Acinetobacter* has been noted for its strong contribution to dissimilarity of microbiome samples from ‘Grenache’ and ‘Carignan’ grape must in Spain from vineyards with different aspects (i.e., flat, east-facing, west-facing), which can reflect variation in temperature and precipitation within a growing region (Portillo et al., 2016). *Paracoccus* has been isolated from winery surfaces (Bokulich et al., 2013), but our work suggests that the vineyard is another source. Recently, it was found on grape leaf surfaces (e.g., ‘Cabernet Sauvignon’, subgenus *Muscadinia*) grown in the same location in France (Montpellier) (Singh et al., 2019). It is difficult to disentangle the influence of the grape must on microbial communities from that of the vineyard environment, given that microbial communities in vineyards have been shown to exhibit annual variation, and vineyard soil and other vegetative parts of grapevines serve as sources of the must microbiome (Belda et al., 2017; Bokulich et al., 2014; Bokulich et al., 2016; Zarraonaindia et al., 2015).

We found no association between must pH and TSS with *Gluconobacter* (*Acetobacteraceae*) and *Tatumella*, as reported in ‘Cabernet Sauvignon’ must for these two organisms (Bokulich et al., 2016) (Fig. 8, Table S6A). *Gluconobacter* is an acetic acid bacterium commonly isolated from grapes and can be found in high abundance (Bokulich et al., 2014), and *Gluconobacter* was negatively associated with ‘Chardonnay’ must pH and TSS in California (Bokulich et al., 2016). *Tatumella* was the only bacterial genus with a significant relationship with titratable acidity (TA). Recently, in another study examining grape musts from red (‘Regent’ and ‘Schwarzriesling’) and white (‘Merzling’, ‘Seyval’, ‘Helios’, and ‘Bacchus’) wine grape cultivars in Germany, *Tatumella* was not a dominant member of the grape musts but it had higher abundances after fermentation (Bubeck et al., 2020). *Lactobacillus* (*Lactobacillaceae*) and *Sphingomonas* (*Sphingomonodaceae*) exhibited slightly negative relationships with total soluble solids (TSS). *Sphingomonas* is a diverse genus, and has been found in high abundances on grape leaves and musts (Bokulich et al., 2014; Leveau & Tech, 2011; Martins et al., 2013).

In our work, the fungal genus *Kazachstania* (*Saccharomycetaceae*) exhibited a slight increase in abundance from 2016 to 2017 (Table S5B), corresponding to annual distinctions in growing season precipitation and GDD. We observed a positive association between *Alternaria* (*Pleosporaceae*), a ubiquitous and diverse fungal genus that contains both plant pathogens and potential post-harvest pathogen, and growing season precipitation, and a negative association for *Hanseniaspora* and *Erysiphe* (*Erysiphaceae*), the causative agent of powdery mildew (Calonnec et al., 2004; Woudenberg, Groenewald & Crous, 2013) (Table S5B). Similarly, *Hanseniaspora* loads (as quantified by qPCR) in grape must from a broad range of cultivars corresponded directly with greater relative humidity or precipitation along a comparable latitudinal gradient (30°S−36°S) in Chile (Jara et al., 2016). The dominant fungal genera (*Alternaria* and *Hanseniaspora*) in the microbial community in our study also overlapped slightly with dominant genera of fungal communities in a recent study of ‘Pinot noir’ must from six wine growing regions in Australia, which included *Alternaria* (*Pleosporaceae*), *Aureobasidium* (*Dothioraceae*)*, Botrytis* (*Sclerotinaceae*), *Cladosporium* (*Cladosporiaceae*), *Epicoccum* (*Pleosporaceae*), *Mycosphaerella* (*Mycosphaerellaeae*), and *Penicillium* (*Trichocomaceae*), and the yeasts *Meyerozyma* (*Debaryomycetaceae*), *Hanseniaspora* and *Saccharomyces* (Liu et al., 2020). With respect to metrics of grape maturity, *Lachancea* (*Saccharomycetaceae*) was positively associated with pH, while *Hanseniaspora, Udeniomyces* (*Cystofilobasidiaceae*)*,* and *Torulaspora* (*Saccharomycetaceae*) associations with pH were negative (Table S5B). Bokulich et al. (2016) reported no association between ‘Cabernet Sauvignon’ and ‘Chardonnay’ must pHs and *Hanseniaspora* in California. Understandably, correlations among variables and the observational nature of this study limit causal links between the variables we measured and abundances of microbial taxa. Finally, metabolic differences between microbial taxa and, likely, differential responses to climate, often occur at the strain level (Albertin et al., 2016; Capece et al., 2005), which we could not resolve using our amplicon sequencing approach.

### Fungal and bacterial communities exhibit distinct geographical patterns

Microbiomes from grape berries, leaves and must can be influenced by many factors, including cultivar, region, vintage and other factors like latitude, longitude, and elevation that can serve as proxies for climate (Belda et al., 2017; Bokulich et al., 2014; Jara et al., 2016; Liu et al., 2020; Portillo et al., 2016; Vitulo et al., 2019). In our study of ‘Pinot noir’ grape must, fungal beta-diversity is correlated with GDD and precipitation (Fig. 7, Table S5). Further, Mantel test results reveal a distance-decay relationship between fungal community structure and geographic distance, or that their dissimilarity increases with increasing distance between vineyards. Such a strong distance-decay relationship suggests either dispersal limitation for fungi or a high degree of adaptation to conditions that correlate with the gradient we observed (Legendre, Borcard & Peres-Neto, 2005). Our vector-fitting analysis (Fig. 7, Table S5) suggests that climate played a role in shaping grape must fungal communities. Similarly, in ‘Pinot noir’ must samples collected from six wine growing regions across Australia, fungal communities varied by region and vintage over two years, as based on their Bray–Curtis dissimilarity of OTUs (operational taxonomic units) (Liu et al., 2020). However, given that GDD and growing season precipitation correlate with the latitudinal gradient in our study, we suggest that dispersal limitation is an important factor shaping these communities, especially as fungal species can have limited dispersal distances often influenced by spore size (Peay & Bruns , 2014). Recent work by Miura et al. (2017) also has displayed the importance of dispersal limitations for microbiome structure on grape leaves and berries. Miura et al. (2017) demonstrated that fungal dissimilarity of communities found on ‘Carmenere’ grape leaves and berries collected from six vineyards in Chile increased with increasing spatial distances (ca. 1–35 km); and that fungal community composition differed among vineyards.

While we detected an association between bacterial community structure and AVA using a PERMANOVA test, our Mantel test results were not significant, indicating the lack of a distance-decay relationship. These results suggest that the bacterial taxa we observed underwent environmental filtering (i.e., the abiotic environment had a role in structuring communities) that may correspond with region, but do not correlate with the geographic distance gradient we observed. Vitulo et al. (2019) also observed that bacterial communities on grape trunk bark were reflective of wine growing region. Similarly, Miura et al. (2017) did not observe a relationship between bacterial dissimilarity (by Bray-Curtis) and geographic distance (ca. 1–35 km) in ‘Carmenere’ grape leaves or berries (six vineyards, Chile); bacterial community composition tended to be similar among vineyards. Miura et al. (2017) suggest that bacteria are not as dispersal limited as fungi as they can remain suspended in the air for longer periods (and thus potentially greater distances) than fungal spores. The correlation between bacterial community structure and fruit maturity metrics that we observed also provides evidence that bacterial communities may be influenced by the maturity of the berry (Table S5).

Our vector-fitting analysis and analysis of beta-diversity using Bray–Curtis distance matrices found that growing season precipitation correlated with bacterial community structure, suggesting that they are sensitive to precipitation (Fig. 6, Table S5). Although bacterial communities did not exhibit a distance-decay relationship, in some cases like in Sonoma, vineyards that were geographically adjacent received different amounts of precipitation. Precipitation also varied considerably between vintages. Together, this variation may have aided in the identification of this correlation in bacterial communities despite the lack of a distance-decay relationship.

Notably, ‘Pinot noir’ grape musts from Monterey exhibited high bacterial diversity and low fungal diversity compared to other AVAs in the 2016 vintage and high fungal diversity compared to other AVAs in the 2017 vintage (Figs. 4 and 5). These high diversity values may correspond to climate, but also with distinctions in grape must characteristics, as suggested by observed values of TA and TSS for the 2016 vintage from Monterey (Fig. 3). Site likely served as a source of the must microbiome and influenced the role of environmental filtering in structuring bacterial communities. The likely role of site as contributor to the must microbiome was revealed in a study on ‘Pinot noir’ must microbial communities from 15 vineyards separated from each other by 5 to 400 km in Australia. Here, Liu et al. (2020) found that both soil properties and soil bacteria had a greater effect than climate on bacterial diversity, even though climate variation by region corresponded to regional distinctions in fungal community diversity.

## Conclusion

Contributing to the growing number of studies appreciating the complexity and diversity grape must microbiomes, our report supports the notion that a predictable, reproducible, high-resolution core microbiome of grape must may not exist. Our findings supported our hypothesis that grape must microbial community structure would be linked to vintage and region, and that must bacterial communities would have dominant members from *Acetobacteraceae* and lactic acid bacteria (in this case, *Lactobacillaceae*). We observed a diverse, variable ‘Pinot noir’ grape must microbiome and variation in taxon distributions across vintages and regions, despite accounting for cultivar, winemaking practices, and winery location. While there are certain patterns that are common; for example, the dominance of *Hanseniaspora* (*Saccharomycetaceae*)*, Tatumella* (*Enterobacteriaceae*), *Gluconobacter*, and *Komagataeibacter* (both *Acetobacteraceae*) and *Lactobacillus (Lactobacillaceae*); at finer taxonomic scales, substantial differences arise. To better understand regional- and vintage-dependent differences in grape must, we propose two avenues for future research: (1) continue to investigate the ecology of microbes in the vineyard and winery to understand the processes that influence the microbial composition of grape must (cf. Liu et al., 2020), and (2) further elucidate the link between early fermentation microbiota and grape must chemistry.

##  Supplemental Information

10.7717/peerj.10836/supp-1 Supplemental Information 1 Additional vineyard and grape must information Additional details about vineyards including elevation, sub-appellation, soil order, soil subgroup, and soil texture. Also includes fruit maturity metrics: total soluble solids (° Brix), pH, and titratable acidity (g tartaric acid L^−1^).

10.7717/peerj.10836/supp-2 Supplemental Information 2 Linear mixed model analysis and tests of mean separation ( *α*= 0.05) as described in Bates et al. (2015) Symbols are as follows: AVA = American Vineyard Area, Vintage = 2016 and 2017, GDD = growing degree days, TA = titratable acidity, TSS = total soluble solids. Symbols for *p*-values are as follows: *p* < 0.10 ‘.’ , *p* < 0.05 ‘*’, *p* < 0.01 ‘**’, *p* < 0.001 ‘***’.

10.7717/peerj.10836/supp-3 Supplemental Information 3 Bacterial core analyses Core bacterial ASVs, defined as having mean abundances above 0.01% in at least 90% of samples, calculated over (A) the entire dataset, (B) the 2016 vintage, and (C) the 2017 vintage. (D) contains variable taxa, defined as having mean abundances above 1% in between 5% and 90% of samples.

10.7717/peerj.10836/supp-4 Supplemental Information 4 Fungal core analyses Core fungal ASVs, defined as having mean abundances above 0.01% in at least 90% of samples, calculated over (A) the entire dataset, (B) the 2016 vintage, and (C) the 2017 vintage. (D) contains variable taxa, defined as having mean abundances above 1% in between 5% and 90% of samples.

10.7717/peerj.10836/supp-5 Supplemental Information 5 Vector-fitting results Vector-fitting results describing associations between (A) bacterial and (B) fungal NMDS coordinates and total soluble solids, pH, titratable acidity, elevation, growing degree days, and growing season precipitation.

10.7717/peerj.10836/supp-6 Supplemental Information 6 Beta binomial regression results Beta binomial regression coefficients, standard errors, *t*-values, *p*-values, and *q*-values for (A) bacterial and (B) fungal genera significantly associated with vintage, growing season precipitation, titratable acidity, pH, and total soluble solids.

10.7717/peerj.10836/supp-7 Supplemental Information 7 Bacterial rarefaction curves Rarefaction curves of bacterial (A) richness, (B) exponential Shannon, and (C) inverse Simpson alpha diversity metrics. Solid line indicates observed values, while dashed line indicates extrapolated values.

10.7717/peerj.10836/supp-8 Supplemental Information 8 Fungal rarefaction curves Rarefaction curves of fungal (A) richness, (B) exponential Shannon, and (C) inverse Simpson alpha diversity metrics. Solid line indicates observed values, while dashed line indicates extrapolated values.

10.7717/peerj.10836/supp-9 Supplemental Information 9 Bacterial differential abundance These plots are the same as Fig. 8, but without the breaks in the *y*-axis (A, B, K and N). Uncorrected relative abundances (percent URAs) of bacterial genera whose beta-binomial abundance models were significantly improved (as determined by a likelihood ratio test) by including (A–F) vintage as a covariate; (G–I) precipitation as a covariate; (J) titratable acidity (g tartaric acid L ^−1^) as a covariate; (K–M) pH as a covariate; (N–O) total soluble solids (TSS; ° Brix) as a covariate. Color of points indicates the respective AVA, as follows: Santa Barbara = red, Monterey = orange, Sonoma = yellow, Mendocino = green, Willamette Valley = blue. Symbols of vintage, as follows: 2016 vintage = circle, 2017 vintage = square.

10.7717/peerj.10836/supp-10 Supplemental Information 10 Fungal differential abundance These plots are the same as Fig. 9, but without the breaks in the *y*-axes on figures (C, D and E). Uncorrected relative abundances (percent URAs) of fungal genera whose beta-binomial abundance models were significantly improved (as determined by a likelihood ratio test) by including (A) vintage as a covariate; (B–D) growing season precipitation as a covariate; (E–H) pH as a covariate; (I) total soluble solids (TSS) as a covariate. Color of points indicates the respective AVA, as follows: Santa Barbara = red, Monterey = orange, Sonoma = yellow, Mendocino = green, Willamette Valley = blue. Symbols of vintage, as follows: 2016 vintage = circle, 2017 vintage = square.

## Additional Information and Declarations

### Data Availability

The following information was supplied regarding data availability: Processed data, including DADA2 output, phyloseq objects, and analysis scripts is available at Github: https://github.com/ianartmor/steenwerth_et_al_2020_peerj. Fungal and bacterial sequences analyzed in this project are available at NCBI: PRJNA642839.
