# Supplementary material for: Fungal and bacterial communities of ‘Pinot noir’ must: effects of vintage, growing region, climate, and basic must chemistry
Source: PeerJ. 2021 Feb 4;9:e10836. doi: 10.7717/peerj.10836 (PMC7868071; doi:10.7717/peerj.10836)
Supplement: Supplemental Information 2 — Symbols are as follows: AVA = American Vineyard Area, Vintage = 2016 and 2017, GDD = growing degree days, TA = titratable acidity, TSS = total soluble solids. Symbols for p-values are as follows: p < 0.10 ‘.’ , p < 0.05 ‘*’, p < 0.01 ‘**’, p < 0.001 ‘***’. [file peerj-09-10836-s002.docx]

Supplemental Table S2. Mixed model results tables

Ian Morelan

11/12/2020

### Must characteristics and Climate

**Must TA AVA/Vintage interaction test**

|  | npar | AIC | BIC | logLik | deviance | Chisq | Df | Pr(>Chisq) |
| --- | --- | --- | --- | --- | --- | --- | --- | --- |
| Must_acidity_reduced_interaction | 8 | 47.62757 | 58.28520 | -15.81378 | 31.62757 | NA | NA | NA |
| Must_acidity_full_model | 12 | 54.04108 | 70.02754 | -15.02054 | 30.04108 | 1.586485 | 4 | 0.8112192 |

**Must TA AVA test**

|  | npar | AIC | BIC | logLik | deviance | Chisq | Df | Pr(>Chisq) |
| --- | --- | --- | --- | --- | --- | --- | --- | --- |
| Must_acidity_AVA | 4 | 51.45505 | 56.78387 | -21.72752 | 43.45505 | NA | NA | NA |
| Must_acidity_reduced_interaction | 8 | 47.62757 | 58.28520 | -15.81378 | 31.62757 | 11.82748 | 4 | 0.0186813 |

**Must TA AVA contrasts**

| term | contrast | estimate | std.error | df | statistic | adj.p.value | signif |
| --- | --- | --- | --- | --- | --- | --- | --- |
| AVA | Mendocino - Monterey | -1.3705994 | 0.4706820 | 22.84919 | -2.9119436 | 0.0550594 | . |
| AVA | Mendocino - Santa Barbara | -0.6760161 | 0.4328291 | 23.20149 | -1.5618545 | 0.5350262 |  |
| AVA | Mendocino - Sonoma | -0.2393494 | 0.3913317 | 23.71245 | -0.6116279 | 0.9718278 |  |
| AVA | Mendocino - Willamette Valley | -0.3633284 | 0.4855299 | 24.21768 | -0.7483131 | 0.9426674 |  |
| AVA | Monterey - Santa Barbara | 0.6945833 | 0.4135245 | 20.94525 | 1.6796666 | 0.4668513 |  |
| AVA | Monterey - Sonoma | 1.1312500 | 0.3698676 | 20.94525 | 3.0585271 | 0.0424205 | * |
| AVA | Monterey - Willamette Valley | 1.0072710 | 0.4706820 | 22.84919 | 2.1400246 | 0.2380109 |  |
| AVA | Santa Barbara - Sonoma | 0.4366667 | 0.3203147 | 20.94525 | 1.3632426 | 0.6564978 |  |
| AVA | Santa Barbara - Willamette Valley | 0.3126877 | 0.4328291 | 23.20149 | 0.7224276 | 0.9491355 |  |
| AVA | Sonoma - Willamette Valley | -0.1239790 | 0.3913317 | 23.71245 | -0.3168129 | 0.9976561 |  |

**Must TA Vintage test**

|  | npar | AIC | BIC | logLik | deviance | Chisq | Df | Pr(>Chisq) |
| --- | --- | --- | --- | --- | --- | --- | --- | --- |
| Must_acidity_Year | 7 | 45.63859 | 54.96402 | -15.81930 | 31.63859 | NA | NA | NA |
| Must_acidity_reduced_interaction | 8 | 47.62757 | 58.28520 | -15.81378 | 31.62757 | 0.0110224 | 1 | 0.9163855 |

**Must pH AVA/Vintage interaction test**

|  | npar | AIC | BIC | logLik | deviance | Chisq | Df | Pr(>Chisq) |
| --- | --- | --- | --- | --- | --- | --- | --- | --- |
| Must_pH_reduced_interaction | 8 | -53.97963 | -43.32199 | 34.98982 | -69.97963 | NA | NA | NA |
| Must_pH_full_model | 12 | -53.14198 | -37.15552 | 38.57099 | -77.14198 | 7.162345 | 4 | 0.1275538 |

**Must pH AVA tes**t

|  | npar | AIC | BIC | logLik | deviance | Chisq | Df | Pr(>Chisq) |
| --- | --- | --- | --- | --- | --- | --- | --- | --- |
| Must_pH_AVA | 4 | -52.00218 | -46.67337 | 30.00109 | -60.00218 | NA | NA | NA |
| Must_pH_reduced_interaction | 8 | -53.97963 | -43.32199 | 34.98982 | -69.97963 | 9.977447 | 4 | 0.0408093 |

**Must pH AVA contrasts**

| term | contrast | estimate | std.error | df | statistic | adj.p.value | signif |
| --- | --- | --- | --- | --- | --- | --- | --- |
| AVA | Mendocino - Monterey | 0.1701224 | 0.0797134 | 23.93719 | 2.1341752 | 0.2388453 |  |
| AVA | Mendocino - Santa Barbara | 0.1117891 | 0.0732257 | 24.23751 | 1.5266381 | 0.5559435 |  |
| AVA | Mendocino - Sonoma | 0.1786641 | 0.0661042 | 24.67263 | 2.7027641 | 0.0823156 | . |
| AVA | Mendocino - Willamette Valley | 0.1883166 | 0.0818632 | 25.04288 | 2.3003800 | 0.1780005 |  |
| AVA | Monterey - Santa Barbara | -0.0583333 | 0.0704355 | 22.30817 | -0.8281807 | 0.9190344 |  |
| AVA | Monterey - Sonoma | 0.0085417 | 0.0629994 | 22.30817 | 0.1355832 | 0.9999174 |  |
| AVA | Monterey - Willamette Valley | 0.0181941 | 0.0797134 | 23.93719 | 0.2282441 | 0.9993523 |  |
| AVA | Santa Barbara - Sonoma | 0.0668750 | 0.0545591 | 22.30817 | 1.2257347 | 0.7368881 |  |
| AVA | Santa Barbara - Willamette Valley | 0.0765275 | 0.0732257 | 24.23751 | 1.0450904 | 0.8319181 |  |
| AVA | Sonoma - Willamette Valley | 0.0096525 | 0.0661042 | 24.67263 | 0.1460187 | 0.9998899 |  |

**Must pH Vintage test**

|  | npar | AIC | BIC | logLik | deviance | Chisq | Df | Pr(>Chisq) |
| --- | --- | --- | --- | --- | --- | --- | --- | --- |
| Must_pH_Year | 7 | -52.50394 | -43.17851 | 33.25197 | -66.50394 | NA | NA | NA |
| Must_pH_reduced_interaction | 8 | -53.97963 | -43.32199 | 34.98982 | -69.97963 | 3.475693 | 1 | 0.0622766 |

**Must TSS AVA/Vintage interaction test**

|  | npar | AIC | BIC | logLik | deviance | Chisq | Df | Pr(>Chisq) |
| --- | --- | --- | --- | --- | --- | --- | --- | --- |
| Must_TSS_reduced_interaction | 8 | 92.39982 | 103.0575 | -38.19991 | 76.39982 | NA | NA | NA |
| Must_TSS_full_model | 12 | 94.60068 | 110.5871 | -35.30034 | 70.60068 | 5.799132 | 4 | 0.2146598 |

**Must TSS AVA test**

|  | npar | AIC | BIC | logLik | deviance | Chisq | Df | Pr(>Chisq) |
| --- | --- | --- | --- | --- | --- | --- | --- | --- |
| Must_TSS_AVA | 4 | 94.14572 | 99.47454 | -43.07286 | 86.14572 | NA | NA | NA |
| Must_TSS_reduced_interaction | 8 | 92.39982 | 103.05745 | -38.19991 | 76.39982 | 9.745906 | 4 | 0.0449324 |

**Must TSS AVA contrasts**

| term | contrast | estimate | std.error | df | statistic | adj.p.value | signif |
| --- | --- | --- | --- | --- | --- | --- | --- |
| AVA | Mendocino - Monterey | 0.4192581 | 0.8752385 | 22.18426 | 0.4790216 | 0.9885174 |  |
| AVA | Mendocino - Santa Barbara | -0.4994919 | 0.8106299 | 22.72812 | -0.6161774 | 0.9710007 |  |
| AVA | Mendocino - Sonoma | -1.3703252 | 0.7404048 | 23.49983 | -1.8507784 | 0.3699976 |  |
| AVA | Mendocino - Willamette Valley | -0.8916667 | 0.9307346 | 24.47175 | -0.9580246 | 0.8710034 |  |
| AVA | Monterey - Santa Barbara | -0.9187500 | 0.7379757 | 19.09142 | -1.2449597 | 0.7261357 |  |
| AVA | Monterey - Sonoma | -1.7895833 | 0.6600655 | 19.09142 | -2.7112208 | 0.0892311 | . |
| AVA | Monterey - Willamette Valley | -1.3109248 | 0.8752385 | 22.18426 | -1.4977914 | 0.5744327 |  |
| AVA | Santa Barbara - Sonoma | -0.8708333 | 0.5716335 | 19.09142 | -1.5234120 | 0.5606193 |  |
| AVA | Santa Barbara - Willamette Valley | -0.3921748 | 0.8106299 | 22.72812 | -0.4837902 | 0.9881008 |  |
| AVA | Sonoma - Willamette Valley | 0.4786585 | 0.7404048 | 23.49983 | 0.6464822 | 0.9656108 |  |

**Must TSS Vintage test**

|  | npar | AIC | BIC | logLik | deviance | Chisq | Df | Pr(>Chisq) |
| --- | --- | --- | --- | --- | --- | --- | --- | --- |
| Must_TSS_Year | 7 | 90.40581 | 99.73124 | -38.20291 | 76.40581 | NA | NA | NA |
| Must_TSS_reduced_interaction | 8 | 92.39982 | 103.05745 | -38.19991 | 76.39982 | 0.0059964 | 1 | 0.9382766 |

**Log Precipitation AVA/Vintage interaction test**

|  | npar | AIC | BIC | logLik | deviance | Chisq | Df | Pr(>Chisq) |
| --- | --- | --- | --- | --- | --- | --- | --- | --- |
| log_Precip_reduced_interaction | 8 | 4.301343 | 14.95898 | 5.849329 | -11.69866 | NA | NA | NA |
| log_Precip_full_model | 12 | 0.409597 | 16.39605 | 11.795201 | -23.59040 | 11.89175 | 4 | 0.0181747 |

**Log Precipitation AVA test**

|  | npar | AIC | BIC | logLik | deviance | Chisq | Df | Pr(>Chisq) |
| --- | --- | --- | --- | --- | --- | --- | --- | --- |
| log_Precip_AVA | 4 | 35.765794 | 41.09461 | -13.882897 | 27.76579 | NA | NA | NA |
| log_Precip_reduced_interaction | 8 | 4.301343 | 14.95898 | 5.849329 | -11.69866 | 39.46445 | 4 | 1e-07 |

**Log Precipitation AVA contrasts**

| Year | term | contrast | estimate | std.error | df | statistic | adj.p.value | signif |
| --- | --- | --- | --- | --- | --- | --- | --- | --- |
| 2016 | AVA | Mendocino - Monterey | 1.6036442 | 0.2792895 | 32.23948 | 5.7418698 | 0.0000211 | *** |
| 2016 | AVA | Mendocino - Santa Barbara | 1.1287731 | 0.2586750 | 33.32791 | 4.3636724 | 0.0010391 | ** |
| 2016 | AVA | Mendocino - Sonoma | 0.2320210 | 0.2362687 | 34.85001 | 0.9820219 | 0.8615209 |  |
| 2016 | AVA | Mendocino - Willamette Valley | -1.0468328 | 0.2991087 | 37.07964 | -3.4998401 | 0.0101622 | * |
| 2016 | AVA | Monterey - Santa Barbara | -0.4748711 | 0.2354769 | 26.04609 | -2.0166354 | 0.2863018 |  |
| 2016 | AVA | Monterey - Sonoma | -1.3716231 | 0.2106170 | 26.04609 | -6.5124060 | 0.0000062 | *** |
| 2016 | AVA | Monterey - Willamette Valley | -2.6504769 | 0.2792895 | 32.23948 | -9.4900688 | 0.0000000 | *** |
| 2016 | AVA | Santa Barbara - Sonoma | -0.8967520 | 0.1823996 | 26.04609 | -4.9164137 | 0.0003722 | *** |
| 2016 | AVA | Santa Barbara - Willamette Valley | -2.1756059 | 0.2586750 | 33.32791 | -8.4105754 | 0.0000000 | *** |
| 2016 | AVA | Sonoma - Willamette Valley | -1.2788538 | 0.2362687 | 34.85001 | -5.4127095 | 0.0000437 | *** |
| 2017 | AVA | Mendocino - Monterey | 1.7814257 | 0.2579520 | 26.17660 | 6.9060348 | 0.0000023 | *** |
| 2017 | AVA | Mendocino - Santa Barbara | 1.1842094 | 0.2354769 | 26.20275 | 5.0289830 | 0.0002738 | *** |
| 2017 | AVA | Mendocino - Sonoma | 0.1529156 | 0.2106170 | 26.24202 | 0.7260365 | 0.9485057 |  |
| 2017 | AVA | Mendocino - Willamette Valley | -0.6406277 | 0.2579520 | 26.30755 | -2.4835146 | 0.1251176 |  |
| 2017 | AVA | Monterey - Santa Barbara | -0.5972163 | 0.2354769 | 26.04609 | -2.5361990 | 0.1131467 |  |
| 2017 | AVA | Monterey - Sonoma | -1.6285101 | 0.2106170 | 26.04609 | -7.7320942 | 0.0000003 | *** |
| 2017 | AVA | Monterey - Willamette Valley | -2.4220534 | 0.2579520 | 26.17660 | -9.3895494 | 0.0000000 | *** |
| 2017 | AVA | Santa Barbara - Sonoma | -1.0312938 | 0.1823996 | 26.04609 | -5.6540344 | 0.0000551 | *** |
| 2017 | AVA | Santa Barbara - Willamette Valley | -1.8248370 | 0.2354769 | 26.20275 | -7.7495370 | 0.0000003 | *** |
| 2017 | AVA | Sonoma - Willamette Valley | -0.7935432 | 0.2106170 | 26.24202 | -3.7677083 | 0.0069157 | ** |

**Log Precipitation Vintage test**

|  | npar | AIC | BIC | logLik | deviance | Chisq | Df | Pr(>Chisq) |
| --- | --- | --- | --- | --- | --- | --- | --- | --- |
| log_Precip_Year | 7 | 51.292060 | 60.61749 | -18.646030 | 37.29206 | NA | NA | NA |
| log_Precip_reduced_interaction | 8 | 4.301343 | 14.95898 | 5.849329 | -11.69866 | 48.99072 | 1 | 2.572e-12 |

**Log Precipitation Vintage contrasts**

| AVA | term | contrast | estimate | std.error | df | statistic | p.value | signif |
| --- | --- | --- | --- | --- | --- | --- | --- | --- |
| Mendocino | Year | 2016 - 2017 | -0.8812384 | 0.1521827 | 22.51846 | -5.790662 | 0.0000073 | *** |
| Monterey | Year | 2016 - 2017 | -0.7034568 | 0.1081488 | 20.78122 | -6.504525 | 0.0000020 | *** |
| Santa Barbara | Year | 2016 - 2017 | -0.8258021 | 0.0883032 | 20.78122 | -9.351897 | 0.0000000 | *** |
| Sonoma | Year | 2016 - 2017 | -0.9603438 | 0.0624398 | 20.78122 | -15.380325 | 0.0000000 | *** |
| Willamette Valley | Year | 2016 - 2017 | -0.4750333 | 0.1521827 | 22.51846 | -3.121467 | 0.0048776 | ** |

**GDD AVA/Vintage interaction test**

|  | npar | AIC | BIC | logLik | deviance | Chisq | Df | Pr(>Chisq) |
| --- | --- | --- | --- | --- | --- | --- | --- | --- |
| GDD_reduced_interaction | 8 | 340.0212 | 350.6788 | -162.0106 | 324.0212 | NA | NA | NA |
| GDD_full_model | 12 | 316.7056 | 332.6920 | -146.3528 | 292.7056 | 31.3156 | 4 | 2.6e-06 |

**GDD AVA test**

|  | npar | AIC | BIC | logLik | deviance | Chisq | Df | Pr(>Chisq) |
| --- | --- | --- | --- | --- | --- | --- | --- | --- |
| GDD_AVA | 4 | 346.1343 | 351.4631 | -169.0672 | 338.1343 | NA | NA | NA |
| GDD_reduced_interaction | 8 | 340.0212 | 350.6788 | -162.0106 | 324.0212 | 14.11314 | 4 | 0.0069426 |

**GDD AVA contrasts**

| Year | term | contrast | estimate | std.error | df | statistic | adj.p.value | signif |
| --- | --- | --- | --- | --- | --- | --- | --- | --- |
| 2016 | AVA | Mendocino - Monterey | -77.23795 | 115.02040 | 23.93803 | -0.6715152 | 0.9606763 |  |
| 2016 | AVA | Mendocino - Santa Barbara | -52.90462 | 105.24834 | 24.14146 | -0.5026646 | 0.9863184 |  |
| 2016 | AVA | Mendocino - Sonoma | -12.23795 | 94.47081 | 24.44610 | -0.1295421 | 0.9999316 |  |
| 2016 | AVA | Mendocino - Willamette Valley | 180.17511 | 116.38087 | 24.95228 | 1.5481505 | 0.5425107 |  |
| 2016 | AVA | Monterey - Santa Barbara | 24.33333 | 103.74197 | 22.91791 | 0.2345563 | 0.9992765 |  |
| 2016 | AVA | Monterey - Sonoma | 65.00000 | 92.78964 | 22.91791 | 0.7005092 | 0.9542839 |  |
| 2016 | AVA | Monterey - Willamette Valley | 257.41306 | 115.02040 | 23.93803 | 2.2379775 | 0.2002631 |  |
| 2016 | AVA | Santa Barbara - Sonoma | 40.66667 | 80.35818 | 22.91791 | 0.5060675 | 0.9859261 |  |
| 2016 | AVA | Santa Barbara - Willamette Valley | 233.07972 | 105.24834 | 24.14146 | 2.2145692 | 0.2082571 |  |
| 2016 | AVA | Sonoma - Willamette Valley | 192.41306 | 94.47081 | 24.44610 | 2.0367462 | 0.2790410 |  |
| 2017 | AVA | Mendocino - Monterey | -198.50000 | 113.64363 | 22.93898 | -1.7466882 | 0.4269680 |  |
| 2017 | AVA | Mendocino - Santa Barbara | -138.50000 | 103.74197 | 22.94320 | -1.3350431 | 0.6730033 |  |
| 2017 | AVA | Mendocino - Sonoma | -40.83333 | 92.78964 | 22.94953 | -0.4400635 | 0.9916817 |  |
| 2017 | AVA | Mendocino - Willamette Valley | 295.50000 | 113.64363 | 22.96008 | 2.6002336 | 0.1034590 |  |
| 2017 | AVA | Monterey - Santa Barbara | 60.00000 | 103.74197 | 22.91791 | 0.5783580 | 0.9769633 |  |
| 2017 | AVA | Monterey - Sonoma | 157.66667 | 92.78964 | 22.91791 | 1.6991840 | 0.4539570 |  |
| 2017 | AVA | Monterey - Willamette Valley | 494.00000 | 113.64363 | 22.93898 | 4.3469219 | 0.0020149 | ** |
| 2017 | AVA | Santa Barbara - Sonoma | 97.66667 | 80.35818 | 22.91791 | 1.2153917 | 0.7427142 |  |
| 2017 | AVA | Santa Barbara - Willamette Valley | 434.00000 | 103.74197 | 22.94320 | 4.1834563 | 0.0029877 | ** |
| 2017 | AVA | Sonoma - Willamette Valley | 336.33333 | 92.78964 | 22.94953 | 3.6246863 | 0.0112320 | * |

**GDD Vintage test**

|  | npar | AIC | BIC | logLik | deviance | Chisq | Df | Pr(>Chisq) |
| --- | --- | --- | --- | --- | --- | --- | --- | --- |
| GDD_Year | 7 | 355.5711 | 364.8966 | -170.7856 | 341.5711 | NA | NA | NA |
| GDD_reduced_interaction | 8 | 340.0212 | 350.6788 | -162.0106 | 324.0212 | 17.54996 | 1 | 2.8e-05 |

**GDD Vintage contrasts**

| AVA | term | contrast | estimate | std.error | df | statistic | p.value | signif |
| --- | --- | --- | --- | --- | --- | --- | --- | --- |
| Mendocino | Year | 2016 - 2017 | -76.73795 | 25.10149 | 21.3399 | -3.057107 | 0.0059113 | ** |
| Monterey | Year | 2016 - 2017 | -198.00000 | 17.75581 | 21.1013 | -11.151281 | 0.0000000 | *** |
| Santa Barbara | Year | 2016 - 2017 | -162.33333 | 14.49756 | 21.1013 | -11.197290 | 0.0000000 | *** |
| Sonoma | Year | 2016 - 2017 | -105.33333 | 10.25132 | 21.1013 | -10.275099 | 0.0000000 | *** |
| Willamette Valley | Year | 2016 - 2017 | 38.58694 | 25.10149 | 21.3399 | 1.537237 | 0.1389313 |  |

###

### Bacterial diversity

**Bacterial richness AVA/Vintage interaction test**

|  | npar | AIC | BIC | logLik | deviance | Chisq | Df | Pr(>Chisq) |
| --- | --- | --- | --- | --- | --- | --- | --- | --- |
| bact_rich_reduced_interaction | 8 | 382.2413 | 392.8989 | -183.1206 | 366.2413 | NA | NA | NA |
| bact_rich_full_model | 12 | 373.7719 | 389.7583 | -174.8859 | 349.7719 | 16.4694 | 4 | 0.0024498 |

**Bacterial richness AVA test**

|  | npar | AIC | BIC | logLik | deviance | Chisq | Df | Pr(>Chisq) |
| --- | --- | --- | --- | --- | --- | --- | --- | --- |
| bact_rich_AVA | 4 | 390.0519 | 395.3807 | -191.0260 | 382.0519 | NA | NA | NA |
| bact_rich_reduced_interaction | 8 | 382.2413 | 392.8989 | -183.1206 | 366.2413 | 15.81066 | 4 | 0.003284 |

**Bacterial richness AVA contrasts**

| Year | term | contrast | estimate | std.error | df | statistic | adj.p.value | signif |
| --- | --- | --- | --- | --- | --- | --- | --- | --- |
| 2016 | AVA | Mendocino - Monterey | -747.36344 | 198.4731 | 43.54735 | -3.7655651 | 0.0042855 | ** |
| 2016 | AVA | Mendocino - Santa Barbara | -391.03011 | 188.0107 | 43.55135 | -2.0798285 | 0.2471239 |  |
| 2016 | AVA | Mendocino - Sonoma | -254.86344 | 176.9308 | 43.55459 | -1.4404697 | 0.6055428 |  |
| 2016 | AVA | Mendocino - Willamette Valley | -405.39863 | 233.4993 | 43.55554 | -1.7361875 | 0.4232227 |  |
| 2016 | AVA | Monterey - Santa Barbara | 356.33333 | 142.1889 | 43.46873 | 2.5060562 | 0.1079211 |  |
| 2016 | AVA | Monterey - Sonoma | 492.50000 | 127.1776 | 43.46873 | 3.8725371 | 0.0031393 | ** |
| 2016 | AVA | Monterey - Willamette Valley | 341.96481 | 198.4731 | 43.54735 | 1.7229780 | 0.4309701 |  |
| 2016 | AVA | Santa Barbara - Sonoma | 136.16667 | 110.1390 | 43.46873 | 1.2363161 | 0.7304393 |  |
| 2016 | AVA | Santa Barbara - Willamette Valley | -14.36852 | 188.0107 | 43.55135 | -0.0764239 | 0.9999919 |  |
| 2016 | AVA | Sonoma - Willamette Valley | -150.53519 | 176.9308 | 43.55459 | -0.8508140 | 0.9129513 |  |
| 2017 | AVA | Mendocino - Monterey | -358.50000 | 155.7601 | 43.47199 | -2.3016161 | 0.1639405 |  |
| 2017 | AVA | Mendocino - Santa Barbara | 154.00000 | 142.1889 | 43.47264 | 1.0830664 | 0.8141982 |  |
| 2017 | AVA | Mendocino - Sonoma | -245.16667 | 127.1776 | 43.47360 | -1.9277503 | 0.3184501 |  |
| 2017 | AVA | Mendocino - Willamette Valley | -194.50000 | 155.7601 | 43.47519 | -1.2487150 | 0.7231842 |  |
| 2017 | AVA | Monterey - Santa Barbara | 512.50000 | 142.1889 | 43.46873 | 3.6043605 | 0.0068131 | ** |
| 2017 | AVA | Monterey - Sonoma | 113.33333 | 127.1776 | 43.46873 | 0.8911422 | 0.8986396 |  |
| 2017 | AVA | Monterey - Willamette Valley | 164.00000 | 155.7601 | 43.47199 | 1.0529011 | 0.8291427 |  |
| 2017 | AVA | Santa Barbara - Sonoma | -399.16667 | 110.1390 | 43.46873 | -3.6242070 | 0.0064403 | ** |
| 2017 | AVA | Santa Barbara - Willamette Valley | -348.50000 | 142.1889 | 43.47264 | -2.4509651 | 0.1212199 |  |
| 2017 | AVA | Sonoma - Willamette Valley | 50.66667 | 127.1776 | 43.47360 | 0.3983930 | 0.9944903 |  |

**Bacterial richness Vintage test**

|  | npar | AIC | BIC | logLik | deviance | Chisq | Df | Pr(>Chisq) |
| --- | --- | --- | --- | --- | --- | --- | --- | --- |
| bact_rich_Year | 7 | 382.4021 | 391.7276 | -184.2011 | 368.4021 | NA | NA | NA |
| bact_rich_reduced_interaction | 8 | 382.2413 | 392.8989 | -183.1206 | 366.2413 | 2.160893 | 1 | 0.1415624 |

**Log Bacterial Exponential Shannon AVA/Vintage interaction test**

|  | npar | AIC | BIC | logLik | deviance | Chisq | Df | Pr(>Chisq) |
| --- | --- | --- | --- | --- | --- | --- | --- | --- |
| log_Bact_Shannon_reduced_interaction | 8 | 91.89439 | 102.55203 | -37.94719 | 75.89439 | NA | NA | NA |
| log_Bact_Shannon_full_model | 12 | 83.85581 | 99.84227 | -29.92791 | 59.85581 | 16.03858 | 4 | 0.0029678 |

**Log Bacterial Exponential Shannon AVA test**

|  | npar | AIC | BIC | logLik | deviance | Chisq | Df | Pr(>Chisq) |
| --- | --- | --- | --- | --- | --- | --- | --- | --- |
| log_bact_Shannon_AVA | 4 | 102.96507 | 108.2939 | -47.48253 | 94.96507 | NA | NA | NA |
| log_bact_Shannon_reduced_interaction | 8 | 91.89439 | 102.5520 | -37.94719 | 75.89439 | 19.07068 | 4 | 0.0007612 |

**Log Bacterial Exponential Shannon AVA contrasts**

| Year | term | contrast | estimate | std.error | df | statistic | adj.p.value | signif |
| --- | --- | --- | --- | --- | --- | --- | --- | --- |
| 2016 | AVA | Mendocino - Monterey | -5.2328183 | 1.1202830 | 43.44542 | -4.6709790 | 0.0002693 | *** |
| 2016 | AVA | Mendocino - Santa Barbara | -2.5207706 | 1.0606997 | 43.49561 | -2.3765167 | 0.1412426 |  |
| 2016 | AVA | Mendocino - Sonoma | -1.8081515 | 0.9975640 | 43.53859 | -1.8125669 | 0.3796512 |  |
| 2016 | AVA | Mendocino - Willamette Valley | -2.5256051 | 1.3154345 | 43.55769 | -1.9199779 | 0.3223654 |  |
| 2016 | AVA | Monterey - Santa Barbara | 2.7120476 | 0.8060708 | 42.54375 | 3.3645279 | 0.0134063 | * |
| 2016 | AVA | Monterey - Sonoma | 3.4246668 | 0.7209716 | 42.54375 | 4.7500716 | 0.0002171 | *** |
| 2016 | AVA | Monterey - Willamette Valley | 2.7072132 | 1.1202830 | 43.44542 | 2.4165440 | 0.1301915 |  |
| 2016 | AVA | Santa Barbara - Sonoma | 0.7126192 | 0.6243797 | 42.54375 | 1.1413233 | 0.7837774 |  |
| 2016 | AVA | Santa Barbara - Willamette Valley | -0.0048344 | 1.0606997 | 43.49561 | -0.0045578 | 1.0000000 |  |
| 2016 | AVA | Sonoma - Willamette Valley | -0.7174536 | 0.9975640 | 43.53859 | -0.7192056 | 0.9509447 |  |
| 2017 | AVA | Mendocino - Monterey | -2.2598938 | 0.8830063 | 42.58740 | -2.5593179 | 0.0965918 | . |
| 2017 | AVA | Mendocino - Santa Barbara | 0.3632926 | 0.8060708 | 42.59602 | 0.4506956 | 0.9911673 |  |
| 2017 | AVA | Mendocino - Sonoma | -1.5210704 | 0.7209716 | 42.60887 | -2.1097507 | 0.2348501 |  |
| 2017 | AVA | Mendocino - Willamette Valley | -1.5249233 | 0.8830063 | 42.63011 | -1.7269677 | 0.4288593 |  |
| 2017 | AVA | Monterey - Santa Barbara | 2.6231864 | 0.8060708 | 42.54375 | 3.2542879 | 0.0179926 | * |
| 2017 | AVA | Monterey - Sonoma | 0.7388234 | 0.7209716 | 42.54375 | 1.0247608 | 0.8425243 |  |
| 2017 | AVA | Monterey - Willamette Valley | 0.7349705 | 0.8830063 | 42.58740 | 0.8323502 | 0.9190497 |  |
| 2017 | AVA | Santa Barbara - Sonoma | -1.8843629 | 0.6243797 | 42.54375 | -3.0179758 | 0.0330267 | * |
| 2017 | AVA | Santa Barbara - Willamette Valley | -1.8882159 | 0.8060708 | 42.59602 | -2.3424939 | 0.1516260 |  |
| 2017 | AVA | Sonoma - Willamette Valley | -0.0038529 | 0.7209716 | 42.60887 | -0.0053441 | 1.0000000 |  |

**Log Bacterial Exponential Shannon Vintage test**

|  | npar | AIC | BIC | logLik | deviance | Chisq | Df | Pr(>Chisq) |
| --- | --- | --- | --- | --- | --- | --- | --- | --- |
| log_bact_Shannon_Year | 7 | 90.17568 | 99.50111 | -38.08784 | 76.17568 | NA | NA | NA |
| log_bact_Shannon_reduced_interaction | 8 | 91.89439 | 102.55203 | -37.94719 | 75.89439 | 0.2812937 | 1 | 0.5958546 |

**Log Bacterial Inverse Simpson AVA/Vintage interaction test**

|  | npar | AIC | BIC | logLik | deviance | Chisq | Df | Pr(>Chisq) |
| --- | --- | --- | --- | --- | --- | --- | --- | --- |
| log_Bact_InvSimp_reduced_interaction | 8 | 81.88539 | 92.54302 | -32.94269 | 65.88539 | NA | NA | NA |
| log_Bact_InvSimp_full_model | 12 | 64.71464 | 80.70109 | -20.35732 | 40.71464 | 25.17075 | 4 | 4.65e-05 |

**Log Bacterial Inverse Simpson AVA test**

|  | npar | AIC | BIC | logLik | deviance | Chisq | Df | Pr(>Chisq) |
| --- | --- | --- | --- | --- | --- | --- | --- | --- |
| log_Bact_InvSimp_AVA | 4 | 90.53078 | 95.85959 | -41.26539 | 82.53078 | NA | NA | NA |
| log_Bact_InvSimp_reduced_interaction | 8 | 81.88539 | 92.54302 | -32.94269 | 65.88539 | 16.64539 | 4 | 0.0022649 |

**Log Bacterial Inverse Simpson AVA contrasts**

| Year | term | contrast | estimate | std.error | df | statistic | adj.p.value | signif |
| --- | --- | --- | --- | --- | --- | --- | --- | --- |
| 2016 | AVA | Mendocino - Monterey | -4.4549011 | 0.7966814 | 42.88045 | -5.5918228 | 0.0000138 | *** |
| 2016 | AVA | Mendocino - Santa Barbara | -1.6246580 | 0.7526394 | 43.14801 | -2.1586140 | 0.2149110 |  |
| 2016 | AVA | Mendocino - Sonoma | -1.1283795 | 0.7058548 | 43.39927 | -1.5986000 | 0.5063218 |  |
| 2016 | AVA | Mendocino - Willamette Valley | -1.4161178 | 0.9273574 | 43.55957 | -1.5270464 | 0.5510002 |  |
| 2016 | AVA | Monterey - Santa Barbara | 2.8302431 | 0.5841023 | 39.02013 | 4.8454577 | 0.0001891 | *** |
| 2016 | AVA | Monterey - Sonoma | 3.3265216 | 0.5224370 | 39.02013 | 6.3673162 | 0.0000015 | *** |
| 2016 | AVA | Monterey - Willamette Valley | 3.0387833 | 0.7966814 | 42.88045 | 3.8143019 | 0.0037682 | ** |
| 2016 | AVA | Santa Barbara - Sonoma | 0.4962785 | 0.4524437 | 39.02013 | 1.0968844 | 0.8070502 |  |
| 2016 | AVA | Santa Barbara - Willamette Valley | 0.2085401 | 0.7526394 | 43.14801 | 0.2770784 | 0.9986562 |  |
| 2016 | AVA | Sonoma - Willamette Valley | -0.2877383 | 0.7058548 | 43.39927 | -0.4076452 | 0.9939825 |  |
| 2017 | AVA | Mendocino - Monterey | -1.1176515 | 0.6398520 | 39.18913 | -1.7467342 | 0.4183424 |  |
| 2017 | AVA | Mendocino - Santa Barbara | 0.1375529 | 0.5841023 | 39.22266 | 0.2354945 | 0.9992882 |  |
| 2017 | AVA | Mendocino - Sonoma | -1.1056643 | 0.5224370 | 39.27277 | -2.1163590 | 0.2336674 |  |
| 2017 | AVA | Mendocino - Willamette Valley | -1.1182589 | 0.6398520 | 39.35583 | -1.7476835 | 0.4177408 |  |
| 2017 | AVA | Monterey - Santa Barbara | 1.2552044 | 0.5841023 | 39.02013 | 2.1489460 | 0.2206573 |  |
| 2017 | AVA | Monterey - Sonoma | 0.0119872 | 0.5224370 | 39.02013 | 0.0229448 | 0.9999999 |  |
| 2017 | AVA | Monterey - Willamette Valley | -0.0006074 | 0.6398520 | 39.18913 | -0.0009492 | 1.0000000 |  |
| 2017 | AVA | Santa Barbara - Sonoma | -1.2432171 | 0.4524437 | 39.02013 | -2.7477829 | 0.0647679 | . |
| 2017 | AVA | Santa Barbara - Willamette Valley | -1.2558117 | 0.5841023 | 39.22266 | -2.1499858 | 0.2201424 |  |
| 2017 | AVA | Sonoma - Willamette Valley | -0.0125946 | 0.5224370 | 39.27277 | -0.0241074 | 0.9999999 |  |

**Log Bacterial Inverse Simpson Vintage test**

|  | npar | AIC | BIC | logLik | deviance | Chisq | Df | Pr(>Chisq) |
| --- | --- | --- | --- | --- | --- | --- | --- | --- |
| log_Bact_InvSimp_Year | 7 | 80.27180 | 89.59723 | -33.13590 | 66.27180 | NA | NA | NA |
| log_Bact_InvSimp_reduced_interaction | 8 | 81.88539 | 92.54302 | -32.94269 | 65.88539 | 0.3864074 | 1 | 0.5341939 |

**Log Bacterial Inverse Simpson Vintage contrasts**

| AVA | term | contrast | estimate | std.error | df | statistic | p.value | signif |
| --- | --- | --- | --- | --- | --- | --- | --- | --- |
| Mendocino | Year | 2016 - 2017 | -1.1241313 | 0.7108870 | 27.05756 | -1.581308 | 0.1254281 |  |
| Monterey | Year | 2016 - 2017 | 2.2131183 | 0.5292162 | 20.50860 | 4.181880 | 0.0004391 | *** |
| Santa Barbara | Year | 2016 - 2017 | 0.6380796 | 0.4321032 | 20.50860 | 1.476683 | 0.1549518 |  |
| Sonoma | Year | 2016 - 2017 | -1.1014161 | 0.3055431 | 20.50860 | -3.604781 | 0.0017140 | ** |
| Willamette Valley | Year | 2016 - 2017 | -0.8262723 | 0.7108870 | 27.05756 | -1.162312 | 0.2552587 |  |

###

### Fungal diversity

**Log Fungal richness AVA/Vintage interaction test**

|  | npar | AIC | BIC | logLik | deviance | Chisq | Df | Pr(>Chisq) |
| --- | --- | --- | --- | --- | --- | --- | --- | --- |
| log_Fung_Rich_reduced_interaction | 8 | -3.124680 | 7.532957 | 9.56234 | -19.12468 | NA | NA | NA |
| log_Fung_Rich_full_model | 12 | -9.881533 | 6.104921 | 16.94077 | -33.88153 | 14.75685 | 4 | 0.005233 |

**Log Fungal richness AVA test**

|  | npar | AIC | BIC | logLik | deviance | Chisq | Df | Pr(>Chisq) |
| --- | --- | --- | --- | --- | --- | --- | --- | --- |
| log_Fung_Rich_AVA | 4 | 10.36662 | 15.695441 | -1.183312 | 2.366623 | NA | NA | NA |
| log_Fung_Rich_reduced_interaction | 8 | -3.12468 | 7.532957 | 9.562340 | -19.124679 | 21.4913 | 4 | 0.000253 |

**Log Fungal richness AVA contrasts**

| Year | term | contrast | estimate | std.error | df | statistic | adj.p.value | signif |
| --- | --- | --- | --- | --- | --- | --- | --- | --- |
| 2016 | AVA | Mendocino - Monterey | 0.0463578 | 0.2129934 | 40.32300 | 0.2176492 | 0.9994792 |  |
| 2016 | AVA | Mendocino - Santa Barbara | -0.0991131 | 0.1998631 | 41.19087 | -0.4959049 | 0.9873118 |  |
| 2016 | AVA | Mendocino - Sonoma | -0.4765537 | 0.1858073 | 42.16802 | -2.5647742 | 0.0956352 | . |
| 2016 | AVA | Mendocino - Willamette Valley | -1.0621025 | 0.2412610 | 43.13082 | -4.4022968 | 0.0006348 | *** |
| 2016 | AVA | Monterey - Santa Barbara | -0.1454709 | 0.1646347 | 32.40787 | -0.8835985 | 0.9008728 |  |
| 2016 | AVA | Monterey - Sonoma | -0.5229116 | 0.1472537 | 32.40787 | -3.5510926 | 0.0098269 | ** |
| 2016 | AVA | Monterey - Willamette Valley | -1.1084603 | 0.2129934 | 40.32300 | -5.2042011 | 0.0000571 | *** |
| 2016 | AVA | Santa Barbara - Sonoma | -0.3774406 | 0.1275255 | 32.40787 | -2.9597278 | 0.0423099 | * |
| 2016 | AVA | Santa Barbara - Willamette Valley | -0.9629894 | 0.1998631 | 41.19087 | -4.8182449 | 0.0001856 | *** |
| 2016 | AVA | Sonoma - Willamette Valley | -0.5855488 | 0.1858073 | 42.16802 | -3.1513768 | 0.0236281 | * |
| 2017 | AVA | Mendocino - Monterey | -0.1443988 | 0.1803482 | 32.63472 | -0.8006664 | 0.9285155 |  |
| 2017 | AVA | Mendocino - Santa Barbara | 0.0744939 | 0.1646347 | 32.68008 | 0.4524801 | 0.9909353 |  |
| 2017 | AVA | Mendocino - Sonoma | -0.2080386 | 0.1472537 | 32.74811 | -1.4127899 | 0.6240752 |  |
| 2017 | AVA | Mendocino - Willamette Valley | -0.6356140 | 0.1803482 | 32.86146 | -3.5243708 | 0.0104172 | * |
| 2017 | AVA | Monterey - Santa Barbara | 0.2188927 | 0.1646347 | 32.40787 | 1.3295662 | 0.6752416 |  |
| 2017 | AVA | Monterey - Sonoma | -0.0636398 | 0.1472537 | 32.40787 | -0.4321778 | 0.9923832 |  |
| 2017 | AVA | Monterey - Willamette Valley | -0.4912153 | 0.1803482 | 32.63472 | -2.7237044 | 0.0719271 | . |
| 2017 | AVA | Santa Barbara - Sonoma | -0.2825325 | 0.1275255 | 32.40787 | -2.2154985 | 0.1996644 |  |
| 2017 | AVA | Santa Barbara - Willamette Valley | -0.7101079 | 0.1646347 | 32.68008 | -4.3132349 | 0.0012341 | ** |
| 2017 | AVA | Sonoma - Willamette Valley | -0.4275755 | 0.1472537 | 32.74811 | -2.9036652 | 0.0479663 | * |

**Log Fungal richness Vintage test**

|  | npar | AIC | BIC | logLik | deviance | Chisq | Df | Pr(>Chisq) |
| --- | --- | --- | --- | --- | --- | --- | --- | --- |
| log_Fung_Rich_Year | 7 | -4.181703 | 5.143728 | 9.090852 | -18.18170 | NA | NA | NA |
| log_Fung_Rich_reduced_interaction | 8 | -3.124680 | 7.532957 | 9.562340 | -19.12468 | 0.9429761 | 1 | 0.3315136 |

**Fungal Exponential Shannon AVA/Vintage interaction test**

|  | npar | AIC | BIC | logLik | deviance | Chisq | Df | Pr(>Chisq) |
| --- | --- | --- | --- | --- | --- | --- | --- | --- |
| fung_Shannon_reduced_interaction | 8 | 90.43900 | 101.09663 | -37.21950 | 74.43900 | NA | NA | NA |
| fung_Shannon_full_model | 12 | 67.23587 | 83.22233 | -21.61794 | 43.23587 | 31.20312 | 4 | 2.8e-06 |

**Fungal Exponential Shannon AVA test**

|  | npar | AIC | BIC | logLik | deviance | Chisq | Df | Pr(>Chisq) |
| --- | --- | --- | --- | --- | --- | --- | --- | --- |
| fung_Shannon_AVA | 4 | 87.88761 | 93.21643 | -39.9438 | 79.88761 | NA | NA | NA |
| fung_Shannon_reduced_interaction | 8 | 90.43900 | 101.09663 | -37.2195 | 74.43900 | 5.448612 | 4 | 0.2442836 |

**Fungal Exponential Shannon AVA contrasts**

| Year | term | contrast | estimate | std.error | df | statistic | adj.p.value | signif |
| --- | --- | --- | --- | --- | --- | --- | --- | --- |
| 2016 | AVA | Mendocino - Monterey | 0.0526418 | 0.8326024 | 43.55556 | 0.0632256 | 0.9999962 |  |
| 2016 | AVA | Mendocino - Santa Barbara | -1.6263156 | 0.7887500 | 43.55556 | -2.0618897 | 0.2549350 |  |
| 2016 | AVA | Mendocino - Sonoma | -2.3044643 | 0.7423115 | 43.55556 | -3.1044436 | 0.0262932 | * |
| 2016 | AVA | Mendocino - Willamette Valley | -2.7067466 | 0.9797206 | 43.55556 | -2.7627740 | 0.0607731 | . |
| 2016 | AVA | Monterey - Santa Barbara | -1.6789573 | 0.5962390 | 43.55556 | -2.8159134 | 0.0536297 | . |
| 2016 | AVA | Monterey - Sonoma | -2.3571061 | 0.5332923 | 43.55556 | -4.4199135 | 0.0005927 | *** |
| 2016 | AVA | Monterey - Willamette Valley | -2.7593884 | 0.8326024 | 43.55556 | -3.3141729 | 0.0151590 | * |
| 2016 | AVA | Santa Barbara - Sonoma | -0.6781487 | 0.4618447 | 43.55556 | -1.4683479 | 0.5879799 |  |
| 2016 | AVA | Santa Barbara - Willamette Valley | -1.0804310 | 0.7887500 | 43.55556 | -1.3698016 | 0.6497708 |  |
| 2016 | AVA | Sonoma - Willamette Valley | -0.4022823 | 0.7423115 | 43.55556 | -0.5419319 | 0.9823614 |  |
| 2017 | AVA | Mendocino - Monterey | -2.2454907 | 0.6531471 | 43.55556 | -3.4379557 | 0.0108273 | * |
| 2017 | AVA | Mendocino - Santa Barbara | -0.2810100 | 0.5962390 | 43.55556 | -0.4713043 | 0.9895455 |  |
| 2017 | AVA | Mendocino - Sonoma | -0.1647139 | 0.5332923 | 43.55556 | -0.3088624 | 0.9979453 |  |
| 2017 | AVA | Mendocino - Willamette Valley | -1.2954762 | 0.6531471 | 43.55556 | -1.9834373 | 0.2909962 |  |
| 2017 | AVA | Monterey - Santa Barbara | 1.9644807 | 0.5962390 | 43.55556 | 3.2947875 | 0.0159675 | * |
| 2017 | AVA | Monterey - Sonoma | 2.0807767 | 0.5332923 | 43.55556 | 3.9017562 | 0.0028746 | ** |
| 2017 | AVA | Monterey - Willamette Valley | 0.9500144 | 0.6531471 | 43.55556 | 1.4545184 | 0.5966959 |  |
| 2017 | AVA | Santa Barbara - Sonoma | 0.1162960 | 0.4618447 | 43.55556 | 0.2518077 | 0.9990775 |  |
| 2017 | AVA | Santa Barbara - Willamette Valley | -1.0144662 | 0.5962390 | 43.55556 | -1.7014424 | 0.4437142 |  |
| 2017 | AVA | Sonoma - Willamette Valley | -1.1307623 | 0.5332923 | 43.55556 | -2.1203422 | 0.2300765 |  |

**Fungal Exponential Shannon Vintage test**

|  | npar | AIC | BIC | logLik | deviance | Chisq | Df | Pr(>Chisq) |
| --- | --- | --- | --- | --- | --- | --- | --- | --- |
| fung_Shannon_Year | 7 | 88.61261 | 97.93804 | -37.3063 | 74.61261 | NA | NA | NA |
| fung_Shannon_reduced_interaction | 8 | 90.43900 | 101.09663 | -37.2195 | 74.43900 | 0.1736098 | 1 | 0.6769233 |

**Fungal Exponential Shannon Vintage contrasts**

| AVA | term | contrast | estimate | std.error | df | statistic | p.value | signif |
| --- | --- | --- | --- | --- | --- | --- | --- | --- |
| Mendocino | Year | 2016 - 2017 | -0.8901375 | 0.8326024 | 29.03704 | -1.0691027 | 0.2938307 |  |
| Monterey | Year | 2016 - 2017 | -3.1882699 | 0.6531471 | 20.49673 | -4.8813967 | 0.0000846 | *** |
| Santa Barbara | Year | 2016 - 2017 | 0.4551681 | 0.5332923 | 20.49673 | 0.8535058 | 0.4032436 |  |
| Sonoma | Year | 2016 - 2017 | 1.2496129 | 0.3770946 | 20.49673 | 3.3137912 | 0.0033813 | ** |
| Willamette Valley | Year | 2016 - 2017 | 0.5211329 | 0.8326024 | 29.03704 | 0.6259084 | 0.5362643 |  |

**Fungal Inverse Simpson AVA/Vintage interaction test**

|  | npar | AIC | BIC | logLik | deviance | Chisq | Df | Pr(>Chisq) |
| --- | --- | --- | --- | --- | --- | --- | --- | --- |
| fung_InvSimp_reduced_interaction | 8 | 82.54947 | 93.20711 | -33.27474 | 66.54947 | NA | NA | NA |
| fung_InvSimp_full_model | 12 | 64.02916 | 80.01561 | -20.01458 | 40.02916 | 26.52031 | 4 | 2.48e-05 |

**Fungal Inverse Simpson AVA test**

|  | npar | AIC | BIC | logLik | deviance | Chisq | Df | Pr(>Chisq) |
| --- | --- | --- | --- | --- | --- | --- | --- | --- |
| fung_InvSimp_AVA | 4 | 79.76126 | 85.09008 | -35.88063 | 71.76126 | NA | NA | NA |
| fung_InvSimp_reduced_interaction | 8 | 82.54947 | 93.20711 | -33.27474 | 66.54947 | 5.211789 | 4 | 0.2662486 |

**Fungal Inverse Simpson AVA contrasts**

| Year | term | contrast | estimate | std.error | df | statistic | adj.p.value | signif |
| --- | --- | --- | --- | --- | --- | --- | --- | --- |
| 2016 | AVA | Mendocino - Monterey | -0.0302493 | 0.7862647 | 43.55556 | -0.0384722 | 0.9999995 |  |
| 2016 | AVA | Mendocino - Santa Barbara | -1.4749447 | 0.7448528 | 43.55556 | -1.9801827 | 0.2925580 |  |
| 2016 | AVA | Mendocino - Sonoma | -1.6889705 | 0.7009988 | 43.55556 | -2.4093770 | 0.1320740 |  |
| 2016 | AVA | Mendocino - Willamette Valley | -1.6556267 | 0.9251951 | 43.55556 | -1.7894892 | 0.3925834 |  |
| 2016 | AVA | Monterey - Santa Barbara | -1.4446953 | 0.5630558 | 43.55556 | -2.5658119 | 0.0948333 | . |
| 2016 | AVA | Monterey - Sonoma | -1.6587211 | 0.5036124 | 43.55556 | -3.2936461 | 0.0160163 | * |
| 2016 | AVA | Monterey - Willamette Valley | -1.6253773 | 0.7862647 | 43.55556 | -2.0672140 | 0.2525992 |  |
| 2016 | AVA | Santa Barbara - Sonoma | -0.2140258 | 0.4361412 | 43.55556 | -0.4907260 | 0.9878264 |  |
| 2016 | AVA | Santa Barbara - Willamette Valley | -0.1806820 | 0.7448528 | 43.55556 | -0.2425741 | 0.9992037 |  |
| 2016 | AVA | Sonoma - Willamette Valley | 0.0333438 | 0.7009988 | 43.55556 | 0.0475661 | 0.9999988 |  |
| 2017 | AVA | Mendocino - Monterey | -2.2815391 | 0.6167967 | 43.55556 | -3.6990130 | 0.0051932 | ** |
| 2017 | AVA | Mendocino - Santa Barbara | -0.7322624 | 0.5630558 | 43.55556 | -1.3005147 | 0.6922831 |  |
| 2017 | AVA | Mendocino - Sonoma | -0.1966567 | 0.5036124 | 43.55556 | -0.3904922 | 0.9948990 |  |
| 2017 | AVA | Mendocino - Willamette Valley | -1.3732980 | 0.6167967 | 43.55556 | -2.2265002 | 0.1893448 |  |
| 2017 | AVA | Monterey - Santa Barbara | 1.5492768 | 0.5630558 | 43.55556 | 2.7515510 | 0.0623830 | . |
| 2017 | AVA | Monterey - Sonoma | 2.0848824 | 0.5036124 | 43.55556 | 4.1398550 | 0.0014067 | ** |
| 2017 | AVA | Monterey - Willamette Valley | 0.9082411 | 0.6167967 | 43.55556 | 1.4725128 | 0.5853542 |  |
| 2017 | AVA | Santa Barbara - Sonoma | 0.5356056 | 0.4361412 | 43.55556 | 1.2280557 | 0.7352386 |  |
| 2017 | AVA | Santa Barbara - Willamette Valley | -0.6410357 | 0.5630558 | 43.55556 | -1.1384940 | 0.7853139 |  |
| 2017 | AVA | Sonoma - Willamette Valley | -1.1766413 | 0.5036124 | 43.55556 | -2.3364025 | 0.1530418 |  |

**Fungal Inverse Simpson Vintage test**

|  | npar | AIC | BIC | logLik | deviance | Chisq | Df | Pr(>Chisq) |
| --- | --- | --- | --- | --- | --- | --- | --- | --- |
| fung_InvSimp_Year | 7 | 80.60058 | 89.92602 | -33.30029 | 66.60058 | NA | NA | NA |
| fung_InvSimp_reduced_interaction | 8 | 82.54947 | 93.20711 | -33.27474 | 66.54947 | 0.051113 | 1 | 0.8211377 |

**Fungal Inverse Simpson Vintage contrasts**

| AVA | term | contrast | estimate | std.error | df | statistic | p.value | signif |
| --- | --- | --- | --- | --- | --- | --- | --- | --- |
| Mendocino | Year | 2016 - 2017 | -0.4475673 | 0.7862647 | 29.03704 | -0.5692324 | 0.5735758 |  |
| Monterey | Year | 2016 - 2017 | -2.6988571 | 0.6167967 | 20.49673 | -4.3756021 | 0.0002780 | *** |
| Santa Barbara | Year | 2016 - 2017 | 0.2951150 | 0.5036124 | 20.49673 | 0.5859962 | 0.5642759 |  |
| Sonoma | Year | 2016 - 2017 | 1.0447464 | 0.3561078 | 20.49673 | 2.9337928 | 0.0080682 | ** |
| Willamette Valley | Year | 2016 - 2017 | -0.1652387 | 0.7862647 | 29.03704 | -0.2101566 | 0.8350132 |  |
