# Supplementary figures and images for: Fungal and bacterial communities of ‘Pinot noir’ must: effects of vintage, growing region, climate, and basic must chemistry
Source: PeerJ. 2021 Feb 4;9:e10836. doi: 10.7717/peerj.10836 (PMC7868071; doi:10.7717/peerj.10836)

A

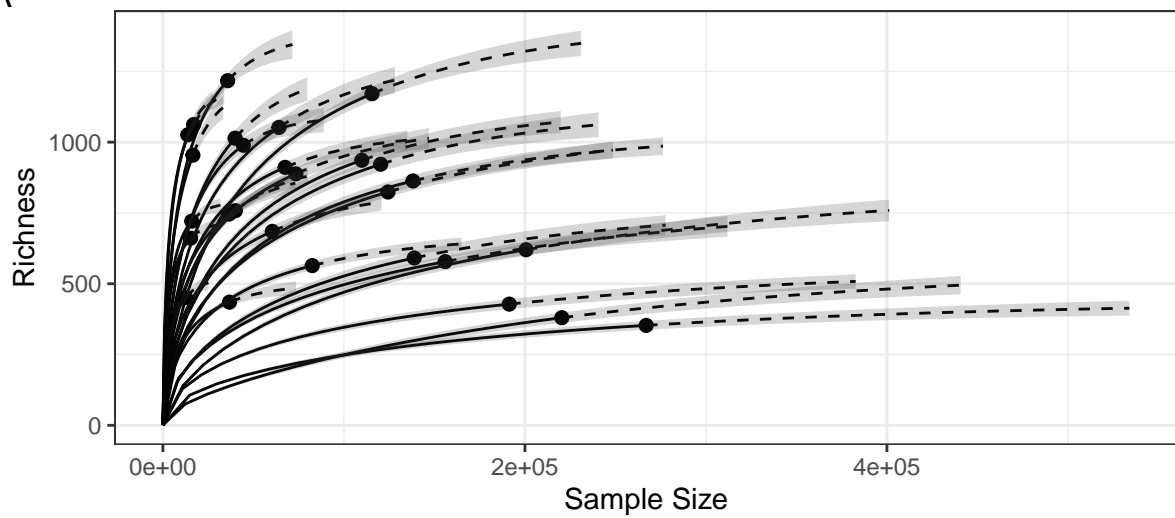

B

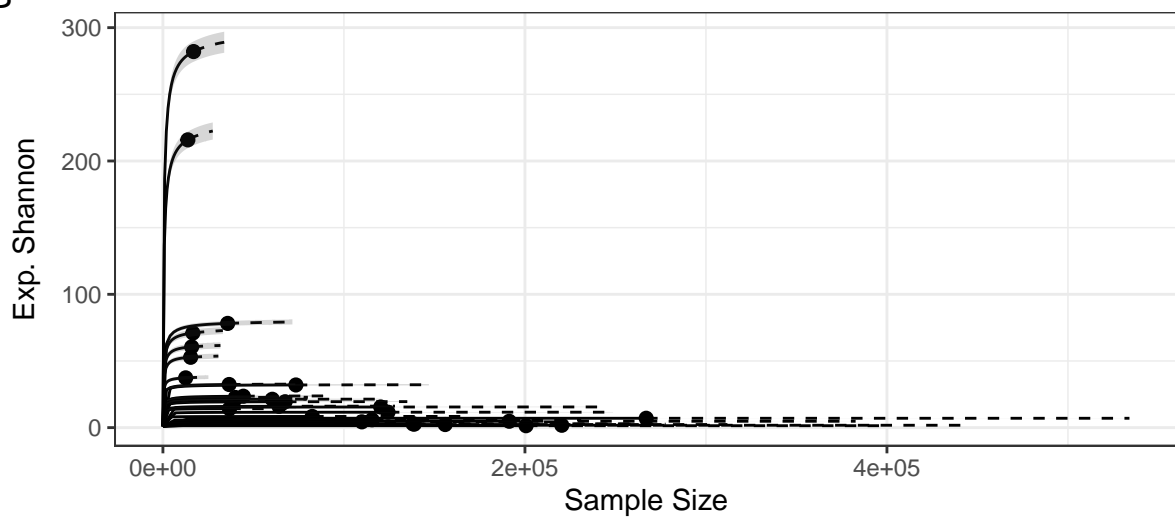

C

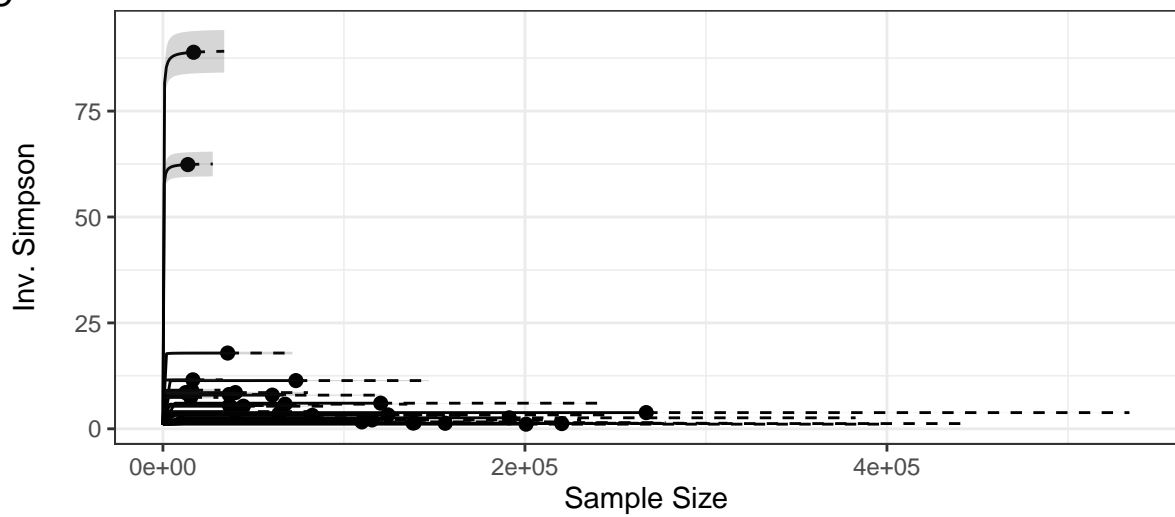

Supplement: Supplemental Information 7 — Rarefaction curves of bacterial (A) richness, (B) exponential Shannon, and (C) inverse Simpson alpha diversity metrics. Solid line indicates observed values, while dashed line indicates extrapolated values. [file peerj-09-10836-s007.pdf]

A

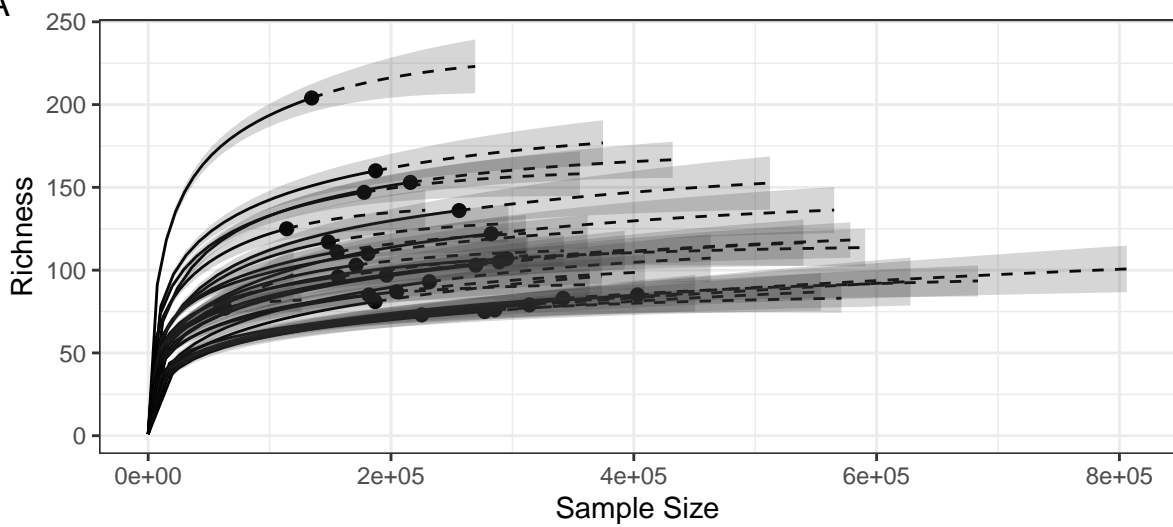

B

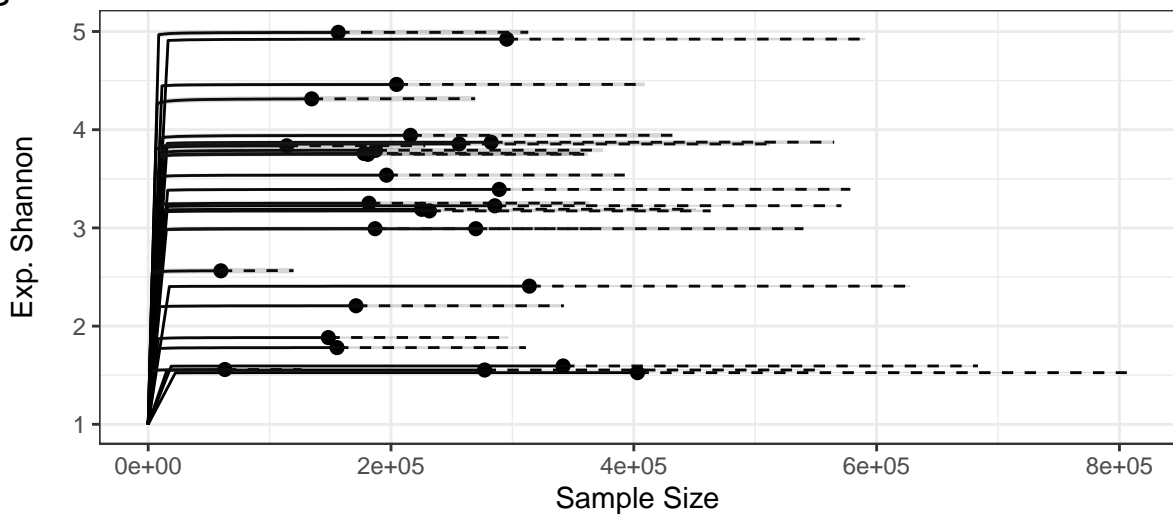

C

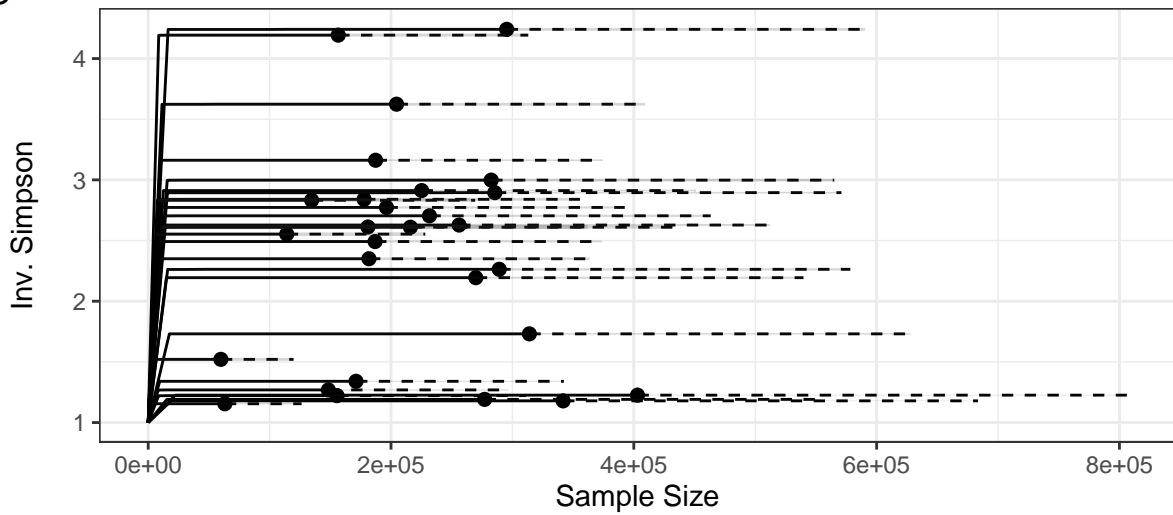

Supplement: Supplemental Information 8 — Rarefaction curves of fungal (A) richness, (B) exponential Shannon, and (C) inverse Simpson alpha diversity metrics. Solid line indicates observed values, while dashed line indicates extrapolated values. [file peerj-09-10836-s008.pdf]

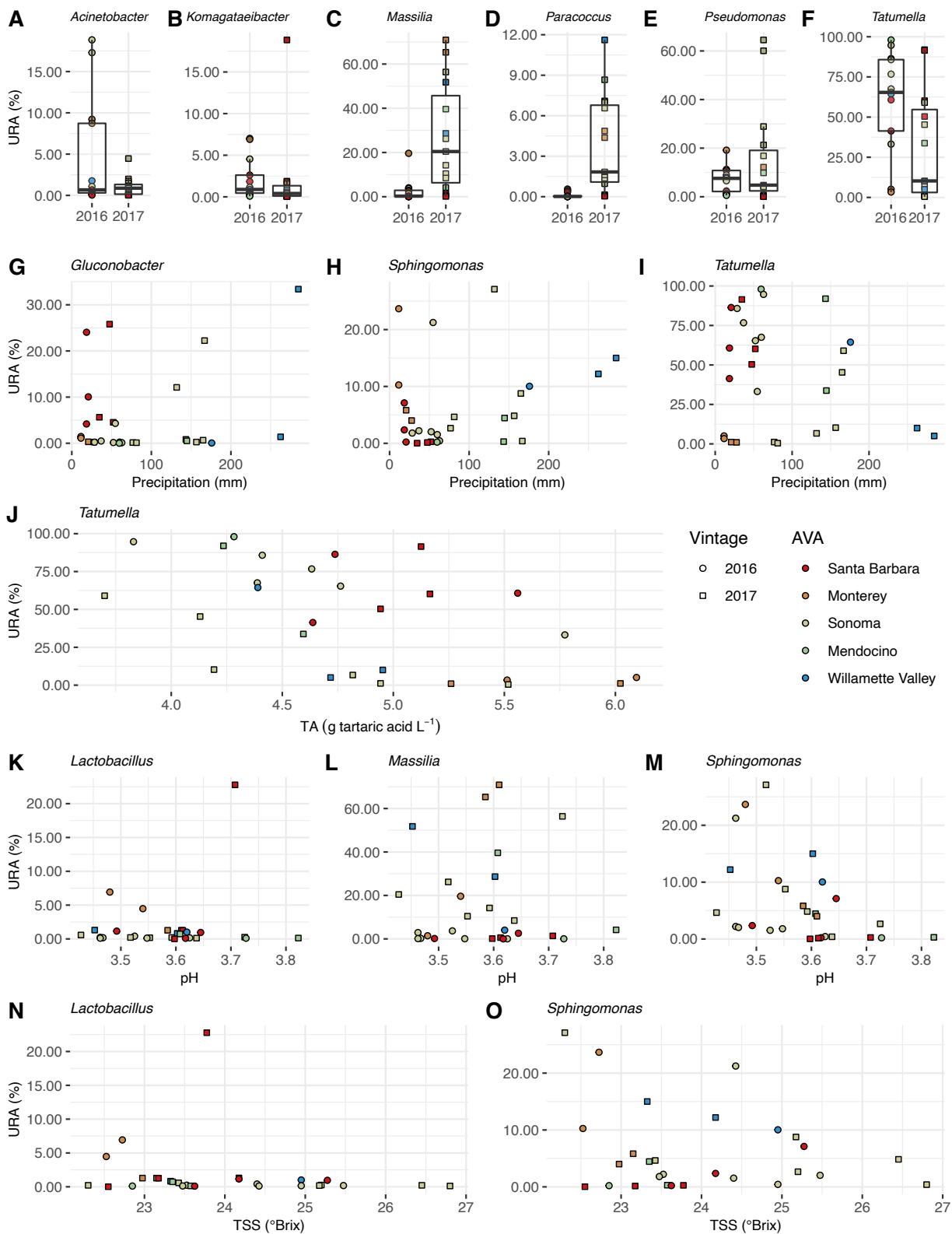

Supplement: Supplemental Information 9 — These plots are the same as Fig. 8, but without the breaks in the y-axis (A, B, K and N). Uncorrected relative abundances (percent URAs) of bacterial genera whose beta-binomial abundance models were significantly improved (as determined by a likelihood ratio test) by including (A–F) vintage as a covariate; (G–I) precipitation as a covariate; (J) titratable acidity (g tartaric acid L −1) as a covariate; (K–M) pH as a covariate; (N–O) total soluble solids (TSS; ° Brix) as a covariate. Color of points indicates the respective AVA, as follows: Santa Barbara = red, Monterey = orange, Sonoma = yellow, Mendocino = green, Willamette Valley = blue. Symbols of vintage, as follows: 2016 vintage = circle, 2017 vintage = square. [file peerj-09-10836-s009.pdf]

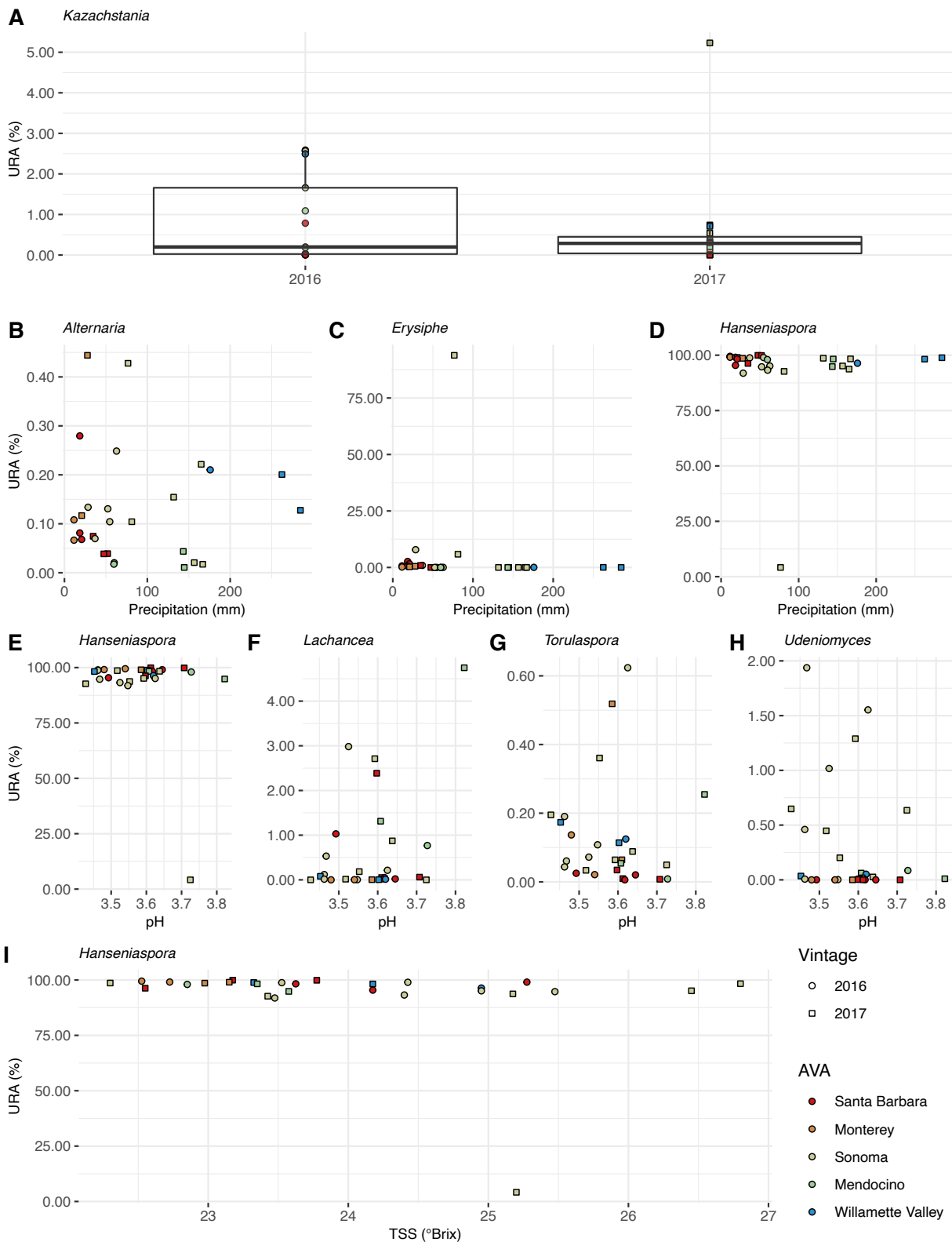

Supplement: Supplemental Information 10 — These plots are the same as Fig. 9, but without the breaks in the y-axes on figures (C, D and E). Uncorrected relative abundances (percent URAs) of fungal genera whose beta-binomial abundance models were significantly improved (as determined by a likelihood ratio test) by including (A) vintage as a covariate; (B–D) growing season precipitation as a covariate; (E–H) pH as a covariate; (I) total soluble solids (TSS) as a covariate. Color of points indicates the respective AVA, as follows: Santa Barbara = red, Monterey = orange, Sonoma = yellow, Mendocino = green, Willamette Valley = blue. Symbols of vintage, as follows: 2016 vintage = circle, 2017 vintage = square. [file peerj-09-10836-s010.pdf]
